# Egg Morphology and Clutch Size in Shrikes (Laniidae) From Historical Oological Collections: Interspecific Patterns and Modest Temporal Change

**DOI:** 10.1002/ece3.74065

**Published:** 2026-07-20

**Authors:** Paweł Pstrokoński, Katarzyna Roguz, Wojciech Wójcik, Martin Päckert, Joanna Rosenberger, Dominika Mierzwa‐Szymkowiak, Magdalena Sepkowska, Jan Lontkowski, Marek Słupek, Michał Chiliński, Krzysztof Damaziak

**Affiliations:** ^1^ Department of Animal Breeding and Nutrition, Institute of Animal Sciences Warsaw University of Life Sciences Warsaw Poland; ^2^ Botanic Garden, Faculty of Biology University of Warsaw Warsaw Poland; ^3^ Evolutionary Ecology of Plants, Department of Biology Marburg University Marburg Germany; ^4^ Senckenberg Natural History Collections Dresden Dresden Germany; ^5^ Division of Poultry Breeding, Faculty of Biology, Institute of Animal Breeding Wroclaw University of Environmental and Life Sciences Wroclaw Poland; ^6^ Museum and Institute of Zoology Polish Academy of Sciences Warsaw Poland; ^7^ Count Antoni Ostrowski Museum in Tomaszow Mazowiecki Tomaszów Mazowiecki Poland; ^8^ Museum of Natural History Wroclaw University Wroclaw Poland; ^9^ Museum of Jacek Malczewski in Radom Radom Poland; ^10^ Faculty of Biology University of Warsaw Warsaw Poland

**Keywords:** avian egg morphology, clutch size variation, life‐history trade‐offs, museum collections, shrikes (Laniidae)

## Abstract

Avian eggs reflect life‐history trade‐offs, and museum collections provide a valuable historical resource for examining how egg morphology and clutch size vary across species, space and time. However, shrikes (Laniidae), despite their ecological distinctiveness and broad distribution, still lack a comparative analysis of egg morphology and clutch size across species. Using historical oological collections spanning 1888–1973, we quantified inter‐ and intraspecific variation in egg morphology and clutch size in four shrike species: red‐backed shrike ( *Lanius collurio* ), woodchat shrike ( *L. senator* ), lesser grey shrike ( *L. minor* ) and great grey shrike ( *L. excubitor* ). Across the historical collection period, clutch size increased over time only in the lesser grey shrike, whereas no robust temporal trend was detected in the other species. Where temporal trends in egg morphology occurred, size‐related traits generally declined, whereas shape descriptors, including sphericity and shape index, remained comparatively stable. Among species, the great grey shrike laid the largest eggs and had the smallest clutches, while the lesser grey and woodchat shrikes had the largest clutches with smaller eggs, consistent with a broad size‐number trade‐off in reproductive allocation. Within species, spatial variation in egg‐size traits was evident, particularly in the red‐backed shrike and woodchat shrike, whereas evidence for a size‐number relationship was weak. Overall, temporal change in shrike reproductive traits was limited and species‐specific, while museum collections proved valuable for reconstructing long‐term patterns of reproductive variation.

## Graphical abstract

Using historical oological collections spanning 1888–1973, we quantified inter‐ and intraspecific variation in egg morphology and clutch size in four shrike species. Among species, egg morphology and clutch size followed a broad size–number trade‐off, with the great grey shrike laying the largest eggs but the smallest clutches, whereas temporal change within species was limited and uneven. Size‐related traits showed greater temporal and spatial variation than shape‐related descriptors, highlighting the value of museum collections for reconstructing long‐term reproductive variation.

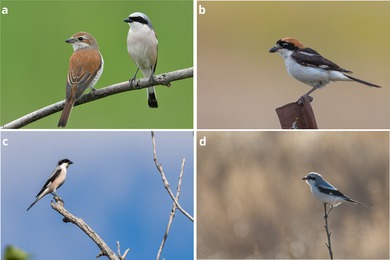

## 1 Introduction

Avian eggs, through their extraordinary diversity in size and shape, provide a unique window into the evolutionary and ecological processes that shape reproductive strategies across bird species. Egg morphology varies markedly across taxa and is shaped by phylogenetic history, developmental constraints and ecological conditions (Sabri et al. 1999; Stoddard et al. 2017). Consequently, eggs can serve as ecological archives, preserving information on reproductive investment, female condition and environmental conditions during egg formation (Hughes 2015; Damaziak and Marzec 2022). Egg morphology is also closely tied to species' life‐history strategies, and multiple evolutionary and ecological pressures act on it (Ousterhout 1980; Rowe et al. 1994; Potti 2008; Evans et al. 2009; Padhi et al. 2013; Shim et al. 2013; Deeming and Reynolds 2015; Birkhead et al. 2019; Ding et al. 2023). Foremost among these allocation patterns is the trade‐off between egg size and clutch size (Blackburn 1991): species producing larger clutches often lay smaller eggs, whereas species investing more resources in individual eggs typically produce fewer eggs (Lack 1947). Conversely, species with intensive parental care or fewer breeding opportunities may invest more resources per offspring, producing larger or energetically more costly eggs (Williams 1994; Christians 2002). Variation in egg traits, including size‐ and shape‐related parameters, reflects both inherited and developmental constraints as well as environmental and maternal effects (Williams 1994; Christians 2002; Stoddard et al. 2017; Birkhead et al. 2019). Egg size is often a repeatable female trait with a genetic component, but it may also vary with female age, condition, food and calcium availability and weather conditions (Williams 1994; Christians 2002; Potti 2008; Hušek et al. 2009; Padhi et al. 2013; Shim et al. 2013; Goławski and Mitrus 2018). Egg shape, in contrast, appears to be more strongly constrained by structural, developmental and evolutionary factors (Stoddard et al. 2017; Birkhead et al. 2019), although spatial and habitat‐related variation in egg shape has also been reported (Bańbura et al. 2018). Because European climates have warmed substantially over the last century (European Environment Agency 2024), long‐term oological datasets can be used to ask whether reproductive traits have changed across historical periods and geographic gradients. Temperature and precipitation may influence egg traits indirectly through female condition, food availability, calcium availability, breeding phenology and the energetic costs of egg formation and incubation (Stevenson and Bryant 2000; Potti 2008; Charmantier and Gienapp 2014; Halupka and Halupka 2017; Goławski and Goławska 2023). However, the expected direction of temporal change is not necessarily straightforward. Warmer conditions may allow females to lay larger eggs if they reduce thermoregulatory costs or improve food availability (Järvinen 1991; Stevenson and Bryant 2000), but egg size may also decline under warming if breeding phenology becomes mismatched with optimal resource conditions (Sanz et al. 2003; Both et al. 2004; Potti 2008). For example, Potti (2008) reported that although females laid larger eggs when experiencing warmer temperatures during the pre‐laying and laying periods, egg volume and breadth declined with increasing spring temperatures across individuals. Thus, environmental change may produce trait‐specific and context‐dependent responses, with egg size expected to be more environmentally responsive than egg shape, which is more strongly constrained by structural, developmental and evolutionary factors (Stoddard et al. 2017; Birkhead et al. 2019). Assessing whether these reproductive traits vary through time, however, requires datasets spanning many decades. Historical specimens preserved in museum collections provide such material, allowing researchers to reconstruct past phenotypes and assess long‐term patterns of variation (Suarez and Tsutsui 2004; Kemp 2015; Holmes et al. 2016; Marini et al. 2020, 2023). Although blown museum eggs do not preserve fresh egg mass or internal components such as yolk, albumen, lipids, hormones, immune factors, or nutrient composition, they retain shell‐based traits that can be measured reliably, including egg length, width, shape, shell pigmentation, shell structure and eggshell mass. These preserved traits allow long‐term, geographic and comparative analyses of egg morphology and reproductive allocation, particularly when specimens are accompanied by information on species identity, locality, collection year and clutch size (Suarez and Tsutsui 2004; Kemp 2015; Holmes et al. 2016; Pstrokoński et al. 2025). Thus, museum egg collections are particularly well suited to investigating broad‐scale variation in egg size, shape and clutch characteristics through time.

Although avian reproductive traits may change over time, not all components of reproduction are expected to do so equally. Some reproductive traits may be less temporally labile than phenological traits because they reflect integrated effects of reproductive allocation, female condition, developmental constraints and species‐specific life‐history strategy (Böhning‐Gaese and Oberrath 1999; Charmantier and Gienapp 2014). By contrast, phenology‐related characteristics, including laying date and migration timing, often show more rapid and plastic responses to environmental change (Charmantier and Gienapp 2014; Halupka and Halupka 2017; Romano et al. 2023). This suggests that long‐term temporal change in egg morphology and clutch size may be detectable, but that different traits may vary in their sensitivity to environmental change. Egg‐size metrics and clutch size may be more environmentally responsive, whereas shape‐related traits may be more strongly constrained by structural and developmental factors, requiring datasets spanning many decades for robust evaluation (Suarez and Tsutsui 2004; Kemp 2015; Holmes et al. 2016; Stoddard et al. 2017; Birkhead et al. 2019).

We focus on shrikes (Laniidae) because they provide a suitable system for comparative analyses of egg morphology and clutch size. They vary considerably in body size and breeding strategies, ranging from sedentary to long‐distance migratory species, while remaining broadly comparable in general breeding ecology as open‐habitat predatory passerines. At the same time, shrikes are ecologically distinctive among passerines because they function as small‐animal predators, feeding on large invertebrates and small vertebrates, and many species are known for prey caching and impaling behaviour. Because egg formation and clutch production depend on female condition and resource availability, this predatory niche may make shrike reproductive investment sensitive to habitat structure, prey availability and seasonal conditions in open landscapes (Degen et al. 1992; Tryjanowski et al. 2003; Hoi et al. 2004; Goławski and Meissner 2008; Morelli et al. 2015). In addition, nest predation may further shape breeding outcomes and reproductive investment (Bechet et al. 1998; Nikolov 2005). Their eggs are also sufficiently distinctive to allow reliable species‐level identification in museum collections, and they differ in both size and shape (Yosef and Zduniak 2004; Goławski and Mitrus 2018). Despite shrikes' broad distribution, a significant knowledge gap remains: a modern comparative analysis of shrike egg morphology and clutch size across species is lacking. At the same time, recent museum‐based work on the red‐backed shrike has shown that long‐term change in eggshell appearance can be reconstructed over more than a century, underscoring the value of shrikes as a model system for archival studies of egg traits (Sulej et al. 2025).

**FIGURE 1:**
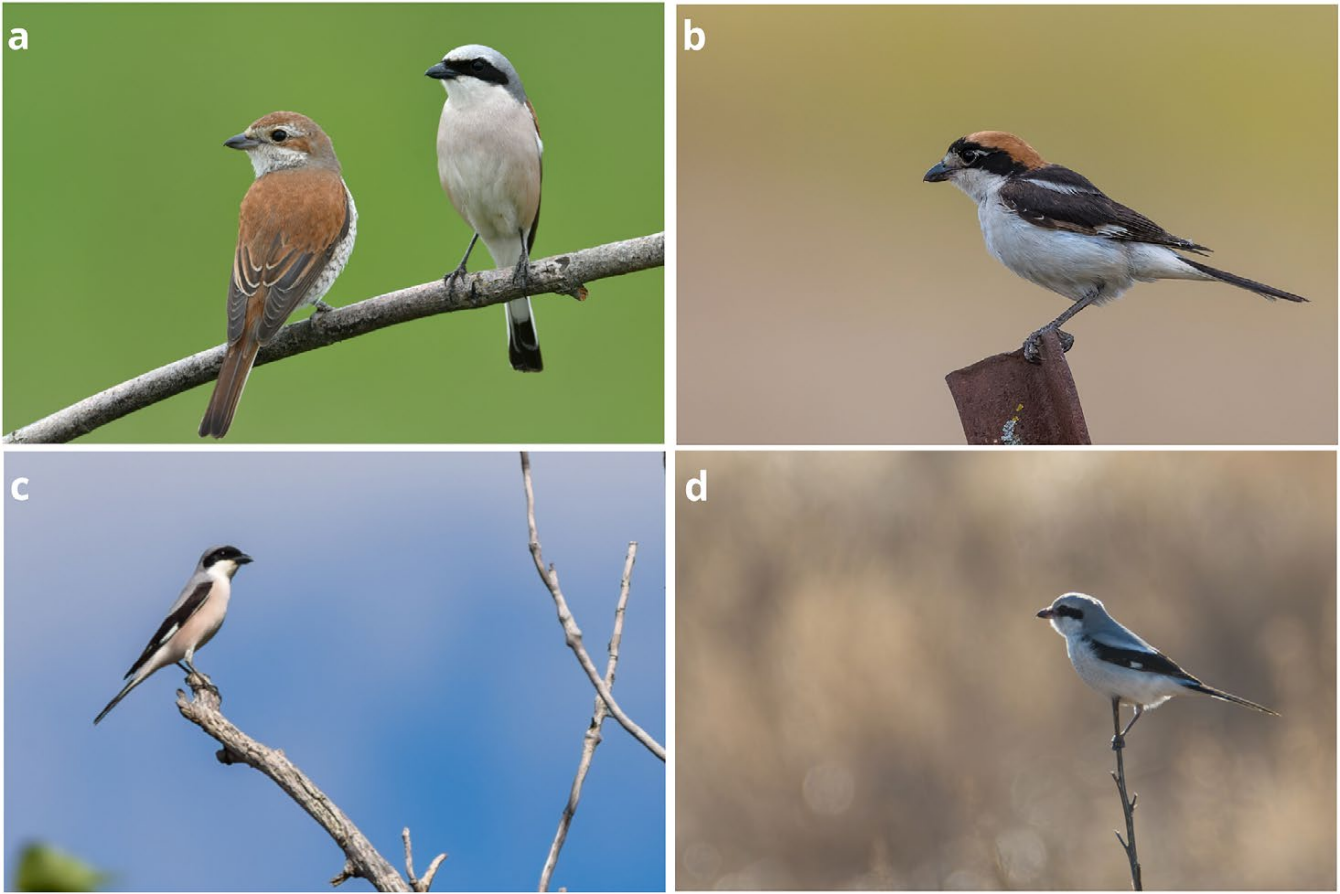
The four shrike species analysed: (a) red‐backed shrike *Lanius collurio* (pair—female [left] and male [right]) (Cezary Korkosz); (b) woodchat shrike *L. senator* (Cezary Korkosz); (c) lesser grey shrike *L. minor* (Maciej Kowalski); (d) great grey shrike *L. excubitor* (Małgorzata Łuczkiewicz). Photos show adult birds.

Previous research on shrike breeding ecology has provided valuable insights into reproductive behaviour and environmental influences, although most studies have focused on single populations or relatively short time frames (typically < 10 years) (Degen et al. 1992; Bechet et al. 1998; Hoi et al. 2004). This leaves unresolved how egg traits and clutch size vary among closely related shrike species and whether such variation is detectable across broader temporal and geographic scales. Historical oological collections allow us to compare interspecific patterns of reproductive investment in shrikes and to assess whether egg traits and clutch size show detectable, although likely weak and species‐specific, temporal and geographic variation. Here, we examine inter‐ and intraspecific variation in egg morphology and clutch size in four shrike species arranged from the smallest to the largest by body mass: the red‐backed shrike ( *Lanius collurio* ; 27.9–29.0 g), woodchat shrike ( *L. senator* ; 34.6–37.4 g), lesser grey shrike ( *L. minor* ; 45.7–47.3 g) and great grey shrike ( *L. excubitor* ; 60.4 g) (Dunning Jr. 2007) (Figure 1). The first three species are long‐distance migrants, whereas the great grey shrike is sedentary or short‐distance migratory across much of its range (BirdLife International 2025a, 2025b, 2025c, 2025d). Using historical oological material collected between 1888 and 1973 from across the species' breeding ranges, we addressed four questions: (1) do egg size, shape and clutch size differ among species; (2) are interspecific differences consistent with body size and a size‐number trade‐off; (3) do egg traits and clutch size vary geographically within species; and (4) is temporal variation in egg morphology and clutch size detectable over the historical sampling period? We predicted that larger‐bodied species would produce larger eggs and smaller clutches, consistent with interspecific reproductive allocation trade‐offs. Within species, we expected geographic variation in egg‐size traits and clutch size, reflecting broad spatial differences in breeding conditions and collection origin. Finally, because egg size and clutch size may respond to environmental and maternal condition, whereas egg shape is more strongly constrained by structural and developmental factors, we predicted that temporal change, if present, would be more evident in size‐related traits than in shape‐related traits.

## 2 Results

### 2.1 Species Differences in Egg Morphology and Clutch Size

**FIGURE 2:**
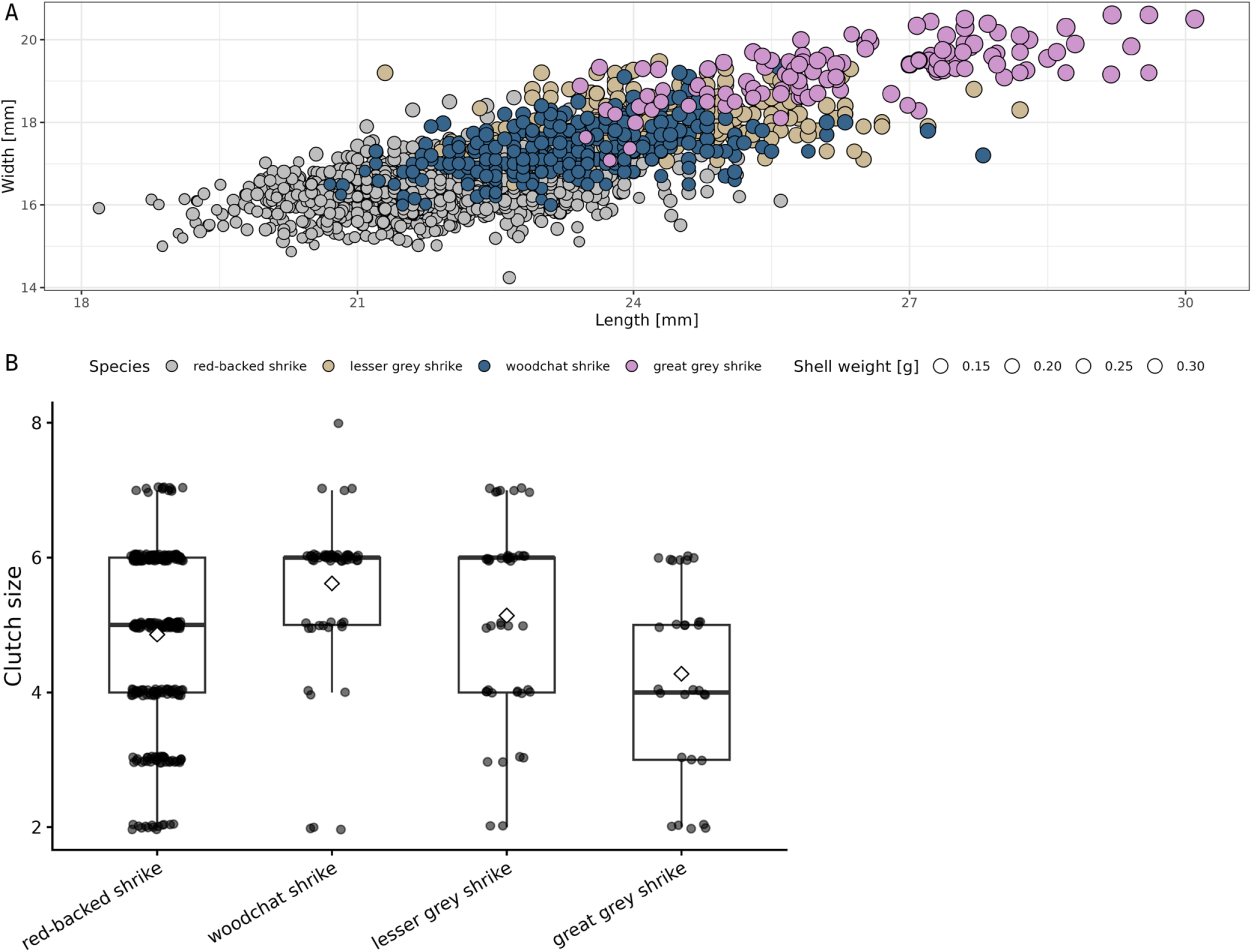
(A) Egg size analysis among shrike species: Red‐backed shrike, woodchat shrike, lesser grey shrike, great grey shrike; egg length (mm), width (mm); dots coloured according to the species and scaled according to the shell weight (g). (B) Comparison of clutch size among four shrike species. Boxplots show the interquartile range, median and whiskers extending to 1.5 × IQR; jittered points represent individual clutches. White diamonds indicate mean values.

**FIGURE 3:**
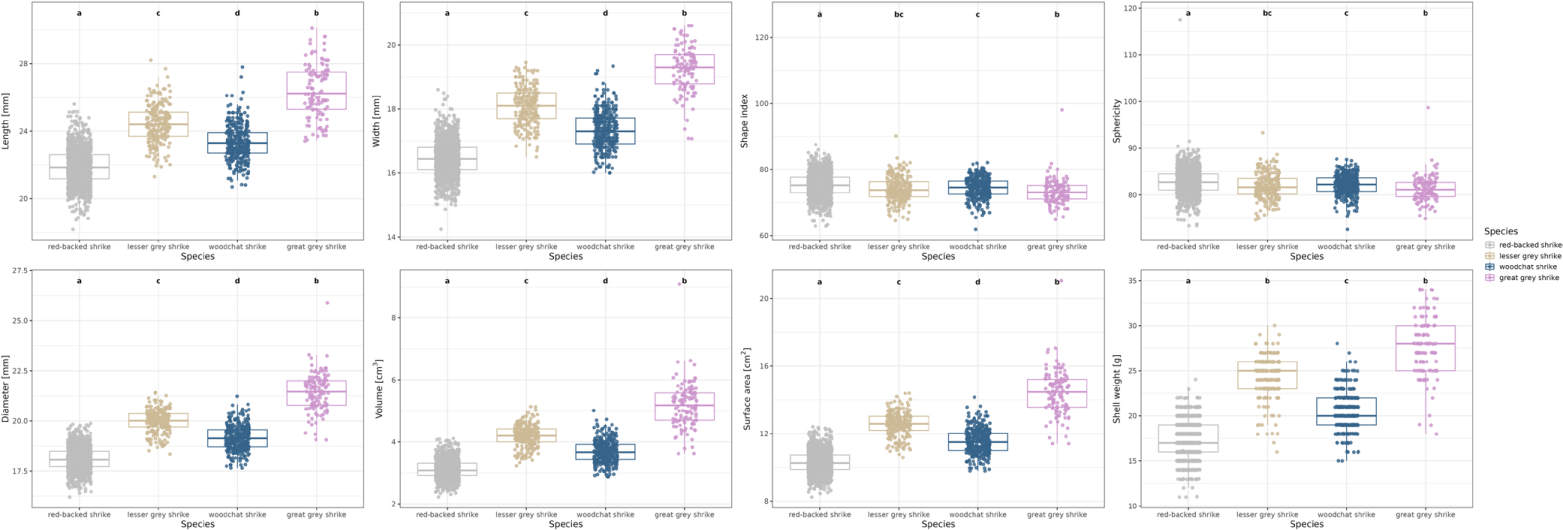
Comparison of egg traits among shrikes: Red‐backed shrike, woodchat shrike, lesser grey shrike and great grey shrike. Panels show: Egg length (mm); egg width (mm); shape index; degree of sphericity; egg diameter (mm); egg volume (cm^3^); egg surface area (mm^2^); shell weight (g). Boxplots show the interquartile range (IQR), median (horizontal line) and whiskers extending to 1.5 × IQR; jittered points indicate individual eggs. Colours distinguish species. Uppercase letters above boxes denote groups from Dunn's post hoc tests with Bonferroni correction (*α* = 0.05); species that do not share a letter differ significantly.

We found significant among‐species differences in egg length and width. The great grey shrike produced the longest eggs (mean ± SD: 26.4 ± 1.56 mm) and the widest eggs (19.3 ± 0.91 mm), whereas the red‐backed shrike produced the shortest eggs (21.9 ± 1.06 mm) and the narrowest eggs (16.5 ± 0.55 mm; Table 1, Figure 2A; *χ* ^2^ = 946, *p* < 0.01 and *χ* ^2^ = 1068, *p* < 0.01, respectively). Similar patterns were observed for egg diameter and volume: the great grey shrike had the largest egg diameter (21.4 ± 0.95 mm) and volume (5.17 ± 0.71 cm^3^), whereas the red‐backed shrike had the smallest diameter (18.1 ± 0.58 mm) and volume (3.11 ± 0.30 cm^3^; Table 1, Figure 3; *χ* ^2^ = 1131, *p* < 0.01 for both traits). Egg surface area was also greatest in the great grey shrike (14.4 ± 1.30 cm^2^) and smallest in the red‐backed shrike (10.3 ± 0.67 cm^2^; Table 1, Figure 3; *χ* ^2^ = 1131, *p* < 0.01). The shape index and degree of egg sphericity also varied significantly among species (Table 1, Figure 3; *χ* ^2^ = 62.7, *p* < 0.01 and *χ* ^2^ = 1068, *p* < 0.01, respectively).

**TABLE 1:** Descriptive statistics for egg traits in four shrike species: Red‐backed shrike, Woodchat shrike, Lesser grey shrike and Great grey shrike.

Trait	Species	*n*	Mean	SD	Median	IQR	SE
Width (mm)	Red‐backed shrike	1313	16.46	0.55	16.44	0.7	0.02
	Great grey shrike	119	19.3	0.91	19.3	0.91	0.08
	Lesser grey shrike	221	18.08	0.58	18.1	0.8	0.04
	Woodchat shrike	337	17.35	0.58	17.3	0.81	0.03
Clutch (*n* eggs)	Red‐backed shrike	1313	5.25	1.13	6	1	0.03
	Great grey shrike	119	4.71	1.26	5	2	0.12
	Lesser grey shrike	221	5.52	1.25	6	1	0.08
	Woodchat shrike	337	5.82	0.84	6	0	0.05
Degree of sphericity	Red‐backed shrike	1313	82.76	2.78	82.71	3.44	0.08
	Great grey shrike	119	81.25	2.81	81.12	3.03	0.26
	Lesser grey shrike	221	81.9	2.79	81.64	3.37	0.19
	Woodchat shrike	337	82.12	2.25	82.23	2.91	0.12
Diameter (mm)	Red‐backed shrike	1313	18.1	0.58	18.06	0.77	0.02
	Great grey shrike	119	21.41	0.95	21.46	1.22	0.09
	Lesser grey shrike	221	19.99	0.57	20.01	0.67	0.04
	Woodchat shrike	337	19.15	0.61	19.13	0.85	0.03
Length (mm)	Red‐backed shrike	1313	21.9	1.06	21.86	1.43	0.03
	Great grey shrike	119	26.39	1.55	26.23	2.2	0.14
	Lesser grey shrike	221	24.44	1.14	24.4	1.43	0.08
	Woodchat shrike	337	23.34	1.05	23.3	1.21	0.06
Shell weight	Red‐backed shrike	1313	16.46	0.55	16.44	0.7	0.02
	Great grey shrike	119	27.69	3.25	28	5	0.3
	Lesser grey shrike	221	24.38	2.2	25	3	0.15
	Woodchat shrike	337	20.38	1.99	20	3	0.11
Shape index	Red‐backed shrike	1313	75.32	3.84	75.22	4.69	0.11
	Great grey shrike	119	73.27	3.87	73.06	4.1	0.35
	Lesser grey shrike	221	74.15	3.8	73.77	4.57	0.26
	Woodchat shrike	337	74.44	3.05	74.56	3.97	0.17
Surface (cm^2^)	Red‐backed shrike	1313	10.3	0.67	10.25	0.87	0.02
	Great grey shrike	119	14.43	1.3	14.47	1.64	0.12
	Lesser grey shrike	221	12.56	0.71	12.58	0.84	0.05
	Woodchat shrike	337	11.53	0.74	11.5	1.02	0.04
Volume (cm^3^)	Red‐backed shrike	1313	3.11	0.3	3.08	0.39	0.01
	Great grey shrike	119	5.17	0.71	5.18	0.88	0.07
	Lesser grey shrike	221	4.19	0.36	4.2	0.42	0.02
	Woodchat shrike	337	3.69	0.36	3.67	0.49	0.02

*Note:* For each studied trait and species, the table reports sample size (*n*), mean, standard deviation (SD), median and standard error of the mean (SE). Traits include clutch size; egg width, diameter, length, degree of sphericity, shape index, shell weight, surface area and volume. Units: Linear dimensions in mm, mass in g, surface area in cm^2^ and volume in cm^3^.

Clutch size differed significantly among species (Table 1, Figure 2B; *χ* ^2^ = 130.0, *p* < 0.01). Mean clutch size did not differ significantly between the lesser grey shrike and the woodchat shrike, but significant differences were observed among the remaining species. The largest clutches were recorded in the woodchat shrike (5.82 ± 0.84 eggs) and the lesser grey shrike (5.52 ± 1.25 eggs), whereas the smallest clutches were recorded in the great grey shrike (4.71 ± 1.26 eggs).

### 2.2 Multivariate Discrimination of Species by Egg Traits

**FIGURE 4:**
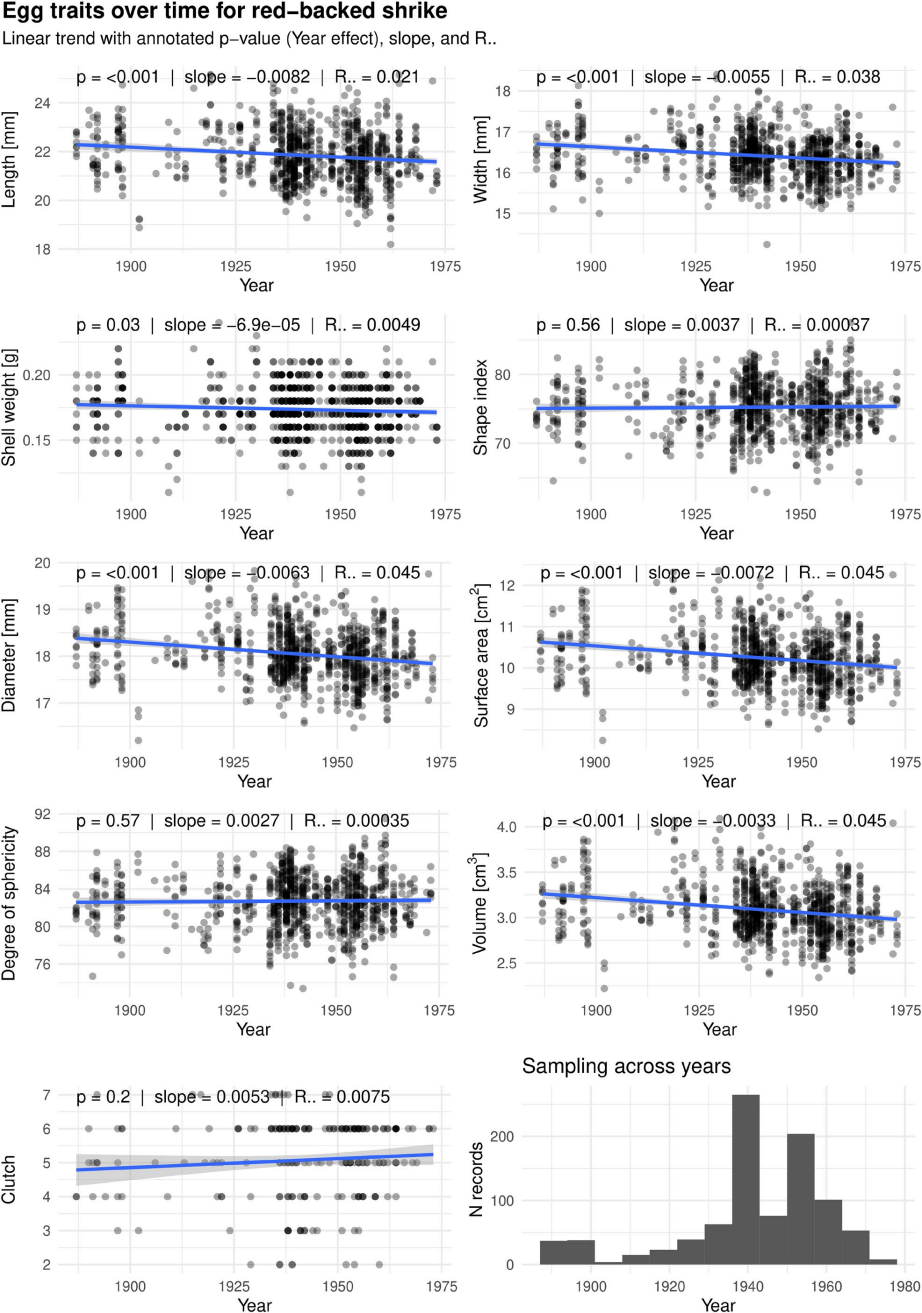
Temporal trends in egg traits and clutch size of red‐backed shrike (1888–1973). Each panel shows annual values with a fitted linear trend (ordinary least squares); panel annotations report the *p*‐value, slope (per year) and *R* ^2^. Studied feature egg length, width, shell weight, surface area, volume, shape index, degree of sphericity and clutch size. The ‘*N* records’ and ‘Sampling across years’ panels summarise temporal sampling effort.

To identify which egg traits best discriminated among shrike species, we performed a linear discriminant analysis. The first three linear discriminants captured most of the interspecific variation in egg morphology. The variables contributing most strongly to species discrimination were egg surface area, eggshell weight and egg volume, followed by egg width (Figure 4, Figure S1). Egg surface area showed the highest positive loadings on both LD1 and LD2. Eggshell weight and egg volume also showed strong, opposing loadings across the discriminant axes, suggesting that species differed primarily along a gradient of egg size and massiveness. Egg width and length contributed moderately, whereas shape index and sphericity had weaker discriminatory value.

### 2.3 Temporal and Spatial Variation in Clutch Size

A GLMM including species, year and mean egg volume, with country fitted as a random intercept, indicated a significant positive effect of collection year on clutch size (*β* = 0.05, 95% CI: 0.02 to 0.08, *p* = 0.001; Table 2). Neither species identity nor mean egg volume significantly predicted clutch size (all *p* ≥ 0.20). Although the coefficient for mean egg volume was negative, consistent in direction with a possible size–number trade‐off, this effect was weak and statistically unsupported. Model fit was modest (marginal *R* ^2^ = 0.042; conditional *R* ^2^ = 0.101), indicating that most variation in clutch size remained unexplained by the fixed effects. The random effect of the country accounted for a small proportion of the total variance (*τ*00 = 0.01, ICC = 0.06). Differences in clutch size among years and countries are presented in Figures S2 and S3 and Tables S1 and S2. As a sensitivity analysis, we refitted the clutch‐size model without the country random intercept. The effect of year remained positive and statistically significant, but model fit was poorer than in the model including country; therefore, the model with country as a random intercept was retained as the main model.

**TABLE 2:** Results of the generalised linear mixed model (GLMM; Conway–Maxwell–Poisson distribution) examining the effects of species, scaled year of egg collection, and mean egg volume on clutch size, with country included as a random intercept.

Predictors	Clutch		
	Estimates	CI	*p*
(Intercept)	1.89	1.61 to 2.16	< 0.001
Great grey shrike	−0.10	−0.32 to 0.11	0.345
Lesser grey shrike	0.09	−0.05 to 0.24	0.212
Woodchat shrike	0.00	−0.12 to 0.13	0.969
Year	0.05	0.02 to 0.08	0.001
Mean volume of egg	−0.05	−0.14 to 0.03	0.202
**Random effects**			
*σ* ^2^	0.20		
*τ*_00 Country_MAIN_	0.01		
ICC	0.06		
*N*_Country_MAIN_	18		
Observations	297		
Marginal *R* ^2^/Conditional *R* ^2^	0.042/0.101		

*Note:* Estimates, 95% confidence intervals (CI), *p*‐values, random‐effect variance components, and model fit statistics are shown.

Species‐specific temporal analyses showed that clutch size increased robustly only in the lesser grey shrike. For this species, clutch size increased over collection years (OLS *β* = 0.03; *p* < 0.01; HC3 *p* = 0.01; Figure S1), and this pattern was supported by sensitivity analyses, including the Theil–Sen estimator (*p* = 0.01) and a GAM indicating mild nonlinearity (edf = 2.74, *p* < 0.01). Kendall's *τ* was borderline (*τ* = 0.28, *p* = 0.059), so we interpret this as an upward temporal trend with possible gentle curvature.

In contrast, the red‐backed shrike showed annual variation in clutch size but no robust temporal trend. Although the linear model estimated a small positive slope (OLS *β* = 0.01; Figure 4), the HC3‐robust test was not significant (*p* = 0.06), the Theil–Sen trend was also non‐significant (*p* = 0.19), and the GAM smooth indicated at most weak nonlinearity (edf = 2.6, *p* = 0.08). The woodchat shrike also showed no robust temporal change in clutch size: the OLS slope was small and borderline (OLS *β* = 0.01; *p* = 0.05), but became non‐significant with HC3 correction (*p* = 0.12), Kendall's *τ* was near zero (*τ* = 0.02, *p* = 0.87), and the Theil–Sen slope was zero (*p* = 0.12). Although the GAM suggested weak nonlinearity (edf = 2.27, *p* = 0.04), the inconsistency among diagnostics argues against a robust temporal trend. No temporal trend in clutch size was detected for the great grey shrike.

### 2.4 Temporal and Spatial Trends in Egg Morphology

Temporal changes in egg morphology differed among species. In the red‐backed shrike, several egg‐size metrics declined over time. Egg width, eggshell weight, diameter, surface area and volume all decreased across collection years, and the robust checks were concordant for these traits (Kendall's *p* ≤ 0.01; Theil–Sen slopes matching OLS; GAM *p* < 0.01; Figure 4). In contrast, clutch size did not show a robust temporal trend in this species, as described above.

For the lesser grey shrike, the main temporal change was detected in clutch size, whereas temporal trends in egg morphology were weaker and less consistent. For the woodchat shrike and great grey shrike, we did not detect robust temporal trends in egg‐size parameters across the diagnostic approaches used. In particular, all temporal analyses for the great grey shrike produced concordant non‐significant results (Figure S1).

Spatial variation in clutch and egg traits was detected among countries, although these patterns should be interpreted cautiously because museum records were unevenly distributed across species, countries and years. Country‐level differences in clutch and egg traits are presented in Figure S3 and Table S2.

### 2.5 Associations Among Clutch Size and Egg Traits

Egg morphology metrics were strongly intercorrelated. Egg length, width, diameter, surface area and volume formed a tightly correlated block of positive associations, with pairwise correlation coefficients typically ranging from |*ρ*| = 0.7–0.9. Shape‐derived indices, including sphericity and shape index, were also correlated with the primary size parameters.

Spearman correlations between clutch size and mean egg traits were uniformly small, and none remained significant after Benjamini–Hochberg false discovery rate correction. These results are consistent with the GLMM analysis, which showed only a weak and statistically unsupported association between mean egg volume and clutch size. Overall, single egg metrics explained little variation in clutch size compared with temporal and spatial structure, although the overall explanatory power of the models remained modest (Figure S4; Table S3).

## 3 Discussion

Using historical egg collections spanning the late nineteenth to mid‐twentieth century, we identified temporal and spatial variation in clutch size and egg morphology across four shrike species. Temporal change in clutch size and egg morphology was limited and species‐specific rather than general across the studied shrikes. Clutch size increased through time in the lesser grey shrike, whereas evidence for temporal change in the remaining species was weak or inconsistent across robustness checks. Where significant temporal trends in egg morphology were detected, they generally involved declines in size‐related traits, most clearly in the red‐backed shrike. By contrast, shape‐related descriptors remained comparatively stable, suggesting that egg size may be more labile than egg shape in this group.

The weak temporal shifts in species‐level means suggest relative stability of clutch size and egg dimensions over the historical period, possibly reflecting shared life‐history constraints rather than strong short‐term environmental lability (Böhning‐Gaese and Oberrath 1999; Charmantier and Gienapp 2014). This interpretation is consistent with earlier and recent work on the red‐backed shrike, which documented long‐term change in egg volume and eggshell appearance, respectively (Tryjanowski et al. 2004; Sulej et al. 2025). In contrast, phenology‐related traits are often more labile and may respond more rapidly to climatic variability and long‐term warming (Charmantier and Gienapp 2014; Halupka and Halupka 2017; Romano et al. 2023). Temporal shifts in egg traits may be subtler and harder to detect, especially in historical datasets with uneven sampling through time (Merilä et al. 2001; Visser et al. 2015). Nevertheless, this relative stability can be overridden by strong environmental pressures, such as organochlorine exposure, causing rapid eggshell thinning and spatial variation in calcium availability affecting eggshell structure (Ratcliffe 1967; Hickey and Anderson 1968; Newton and Bogan 1974; Burnett et al. 2013; Gosler and Wilkin 2017). Together, these findings suggest that temporal change in shrike egg traits can occur, but it is modest relative to broader interspecific and spatial variation.

In addition to temporal patterns, we also found spatial within‐species variation in egg‐size traits and clutch size, particularly in the two smallest species, the red‐backed shrike and the woodchat shrike, whereas egg shape did not vary geographically in any species. This pattern suggests that size‐related egg traits may be more responsive to local conditions than shape‐related descriptors. Spatial variation in egg size may reflect environmental heterogeneity, including differences in habitat quality or climate (Bańbura et al. 2018), although the available museum material does not allow these mechanisms to be tested directly. This interpretation is consistent with previous work on the red‐backed shrike. No differences in egg dimensions were detected among clutch‐size classes, although rainfall before laying influenced within‐clutch repeatability of egg volume, suggesting selective rather than uniform environmental effects on egg traits (Goławski and Mitrus 2018). By contrast, no effects of temperature or rainfall on clutch size, egg volume, within‐clutch variation, or hatchability were found in another red‐backed shrike population, indicating that weather‐related responses may vary among populations or study contexts (Goławski 2008). The lack of comparable spatial variation in the lesser grey shrike further indicates that these patterns are not uniform across species. More broadly, temporal and spatial variation in egg traits may reflect a combination of population structure, environmental heterogeneity and reproductive constraints, although the relative importance of these factors cannot be resolved with museum material alone.

Among species, the observed differences in egg morphology and clutch size were broadly consistent with body size and reproductive allocation. The great grey shrike, the largest species in the dataset, laid the largest and heaviest eggs but had the smallest clutch size, whereas the red‐backed shrike laid the smallest and lightest eggs. This pattern is consistent with a classic interspecific size‐number trade‐off, in which greater investment per offspring is associated with reduced clutch size (Saether 1985; Blackburn 1991; Martin et al. 2006). The woodchat and lesser grey shrikes, despite producing smaller eggs, had the largest clutches, reinforcing this general pattern. In contrast, within‐species associations between clutch size and egg traits were weak, indicating that this trade‐off was not strongly supported at the intraspecific level.

These findings should be interpreted in light of the limitations of museum oological material. Although historical collections provide access to large numbers of specimens across broad temporal and geographic ranges, they lack information on key reproductive and environmental variables, including female age, breeding conditions and reproductive success, and they may also reflect collector bias (Thompson and Birkhead 2020). Clutch size was obtained from museum‐label information; records with incomplete or ambiguous data were excluded. In addition, the most recent eggs in our material date to 1973, leaving a gap of more than 50 years between the historical dataset and present‐day populations. Addressing this gap is challenging as large oological collections were assembled under historical conditions that are no longer replicable under modern legal and ethical standards. Although our museum dataset ends in 1973, contemporary field‐based data show that clutch size and egg dimensions can be collected without adding new specimens to oological collections. Available post‐1973 studies provide useful reference points for some shrike species, although these data remain scattered among local populations and differ in sampling design, geographic context and whether first clutches, replacement clutches, or all breeding attempts were included. For example, in a 23‐year field study of the red‐backed shrike in east‐central Poland, mean clutch size was 5.5 eggs and mean egg volume was 3.19 cm^3^, values close to those observed in our historical material (Goławski and Goławska 2023). Similarly, first clutches of the lesser grey shrike in Central Slovakia averaged 5.7–5.8 eggs, comparable to our mean clutch size of 5.52 eggs (Krištín et al. 2000). In contrast, a western Polish population of the great grey shrike showed a higher mean clutch size of 6.6 eggs and larger eggs, with mean dimensions of 27.1 × 19.9 mm (Antczak et al. 2004), whereas a recent woodchat shrike population near the southern edge of its breeding range in northern Israel showed a lower mean clutch size of 4.63 eggs (Bloche and Sapir 2024). These differences may reflect local ecological conditions, population‐specific reproductive performance, breeding attempt type, or methodological differences rather than directional temporal change. Therefore, we did not merge these contemporary data with our historical dataset, but we identify the integration of museum collections with standardised contemporary field measurements as an important future direction. Despite these limitations, museum collections remain highly valuable for reconstructing long‐term reproductive variation and for providing a historical baseline against which contemporary data can be compared.

In summary, historical oological collections revealed clear among‐species differences in egg morphology and clutch size, but only limited and uneven temporal change within species. Size‐related traits showed greater temporal and spatial variation than shape‐related descriptors, while evidence for a within‐species size–number relationship was weak. Overall, these results highlight both the value of museum material for comparative studies of avian reproduction and the need for contemporary data to place historical patterns in a modern ecological context.

## 4 Methods

### 4.1 Egg Measurements

We measured a total of 1990 eggs from 409 clutches across four species of the shrike family, Laniidae. This included 1313 eggs from 284 clutches of the red‐backed shrike, 337 eggs from 60 clutches of the woodchat shrike, 221 eggs from 43 clutches of the lesser grey shrike, and 119 eggs from 22 clutches of the great grey shrike. We determined clutch size parameters based on information provided on museum labels. We excluded nests with incomplete or uncertain data, such as ambiguous collection year, location, or maternal origin. This also ensured that no multiple clutches from the same female were included.

**FIGURE 5:**
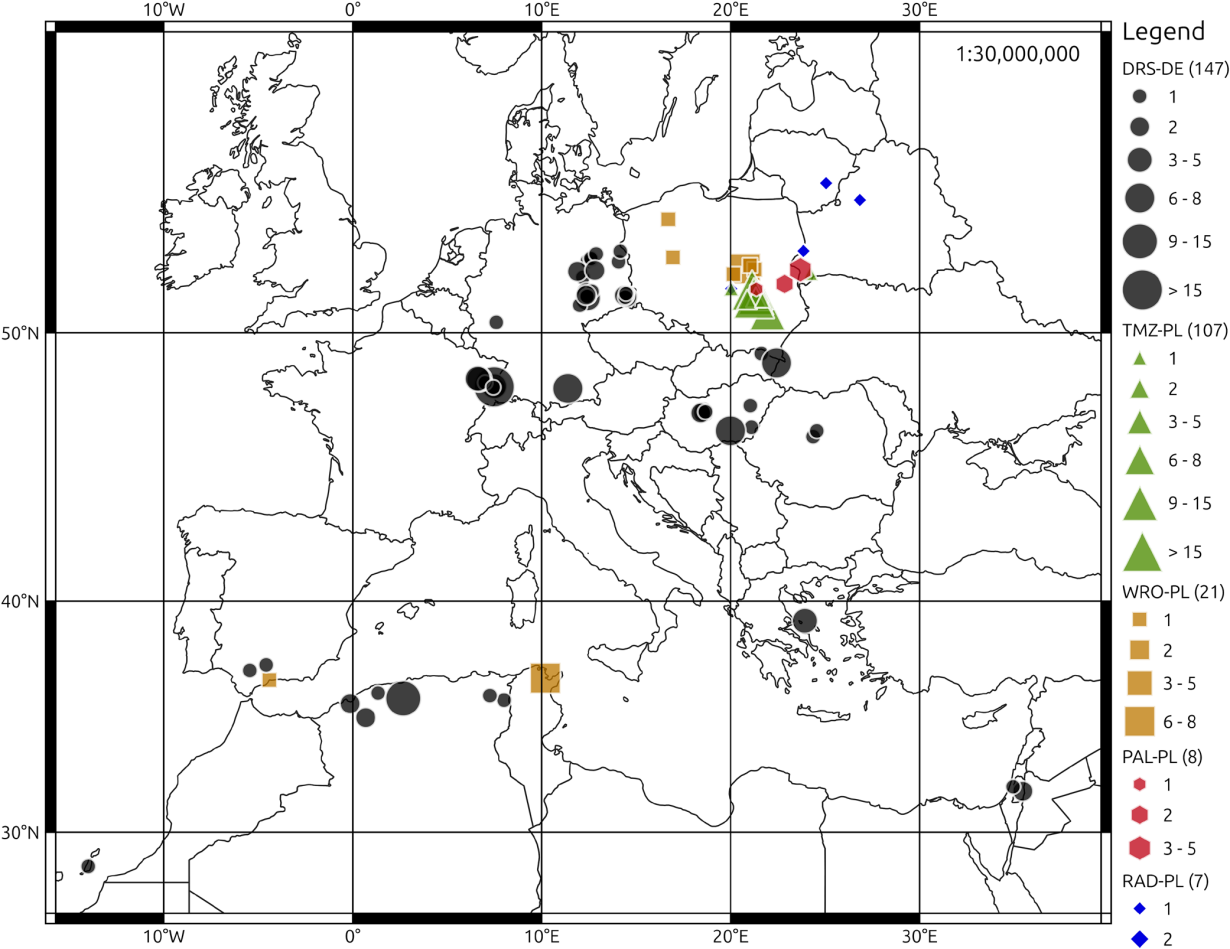
Geographic distribution of 290 clutches of red‐backed shrike, woodchat shrike, lesser grey shrike and great grey shrike collected between 1888 and 1973 from six oological collections (BDZ‐PL, TMZ‐PL, PAL‐PL, WRO‐PL, RAD‐PL, DRS‐DE). Numbers in parentheses represent the total number of clutches with determined location held in each collection. Different symbol shapes and colours denote individual collections (see legend), and symbol size is proportional to the number of clutches per locality.

We collected egg measurements from six locations housing oological collections: five in Poland and one in Germany. These included: (1) Świętokrzyski National Park in Bodzentyn, Poland (BDZ‐PL); (2) Count Antoni Ostrowski Museum in Tomaszów Mazowiecki, Poland (TMZ‐PL); (3) Museum and Institute of Zoology Polish Academy of Science, Research Station in Palmiry, Poland (PAL‐PL); (4) Museum of Natural History at the University of Wrocław, Poland (WRO‐PL); (5) Jacek Malczewski Museum in Radom, Poland (RAD‐PL); and (6) Senckenberg Natural History Collections in Dresden, Germany (DRS‐DE). Based on the information provided on museum labels, we determined collection localities for 290 clutches collected between 1888 and 1973; clutches with incomplete or ambiguous data, or cases where the collection site could not be reliably identified, were excluded (Figure 5). All eggs used in this study were blown specimens stored in closed cabinets without exposure to light. Measurements were taken after temporarily removing the eggs from the display cases. We measured each egg's maximum length and width to the nearest 0.01 mm with a Mitutoyo electronic sliding calliper (Kanagawa, Japan) and determined shell weight with a Kern & Sohn GmbH balance (Balingen, Germany). All measurements were taken by a single researcher (KD), ensuring consistency and eliminating inter‐observer variability.

The dimensions obtained—length (*L*) and width (*W*)—were used to calculate five geometrical parameters with the use of the following formulas:

The egg shape index (SI) (Sarica and Erensayin 2004):

(1) $$\mathrm{SI}=\left(W/L\right)\times 100;$$

The geometric mean diameter of eggs (Dg) (Mohsenin 1970):

(2) $$\mathrm{Dg}={\left(L\times W^{2}\right)}^{⅓};$$

The surface area of eggs (*S*) (Mohsenin 1970; Baryeh and Mangope 2003):

(3) $$S=\pi \times {\mathrm{Dg}}^{2};$$

The degree of sphericity of eggs (*Φ*):

(4) $$\Phi =\left(\mathrm{Dg}/L\right)\times 100;$$

and the volume of eggs (*V*) (Hoyt 1979; Severa et al. 2013; Kumbar et al. 2016):

(5) $$V=\left(\pi /6\right)\times L\times W^{2}.$$

### 4.2 Statistical Analysis

Initially, we assessed the normality of our data using the Shapiro–Wilk test. Given that the data did not meet the normality assumption, even after attempting appropriate transformations, we proceeded with the Kruskal‐Wallis ANOVA test (hereafter KW) to identify differences in egg size parameters.

We used generalised linear mixed models (GLMMs) with a Conway–Maxwell–Poisson distribution to evaluate the effects of species, year of egg collection and egg size on clutch size. Country was included as a random intercept. We analysed determinants of clutch size at the clutch level, using only averages of egg traits per clutch. Because egg size metrics were highly collinear (length, width, diameter, surface area, volume), we adopted a ‘one variable per construct’ rule and retained mean egg volume as the single size proxy. Fixed effects were year, species and mean egg volume. Models were fitted in R using the package glmmTMB (Brooks et al. 2017). To evaluate whether the COM‐Poisson distribution was appropriate, we fitted alternative GLMMs with identical fixed and random effects structures but assuming Poisson and negative binomial (nbinom2) error distributions and compared models using Akaike's information criterion (AIC). The nbinom1 model failed to converge and was therefore not considered further. Model diagnostics were performed using the package DHARMa (Hartig 2024), based on simulated scaled residuals. We tested for dispersion, zero inflation and deviations from a uniform distribution of residuals and inspected residual plots visually. Residual diagnostics using DHARMa (dispersion = 0.97, *p* = 0.98; zero‐inflation test ratioObsSim ≈ 1, *p* = 1; some deviations from uniformity) and the overdispersion check in the performance package (Lüdecke et al. 2024) (dispersion ratio = 0.96, *p* = 0.95) indicated no overdispersion or zero inflation and a mild tendency towards underdispersion. We therefore modelled clutch size using a GLMM with a COM‐Poisson distribution and log link in the glmmTMB package (Brooks et al. 2017), retaining species, year and mean egg volume as fixed effects and including country as a random intercept.

For each response variable (egg traits), we quantified temporal change within the analysed subset by fitting an ordinary least squares (OLS) linear regression of egg traits on a calendar year. For traits showing significant temporal differences, we performed additional analyses. We repeated the tests using heteroscedasticity‐robust standard errors and a robust Theil–Sen estimator; where results remained unclear, we applied a generalised additive model.

All statistical analyses were performed using R version 4.1.2 (R Core Team 2021).

## Author Contributions

**Paweł Pstrokoński:** conceptualization (lead), data curation (equal), investigation (lead), methodology (equal), writing – original draft (lead). **Katarzyna Roguz:** data curation (equal), formal analysis (lead), methodology (equal), visualization (lead), writing – original draft (lead). **Wojciech Wójcik:** data curation (equal), investigation (lead), writing – review and editing (equal). **Martin Päckert:** investigation (equal), resources (equal), writing – review and editing (equal). **Joanna Rosenberger:** investigation (equal), resources (equal), writing – review and editing (equal). **Dominika Mierzwa‐Szymkowiak:** investigation (equal), resources (equal), writing – review and editing (equal). **Magdalena Sepkowska:** investigation (equal), resources (equal), writing – review and editing (equal). **Jan Lontkowski:** investigation (equal), resources (equal), writing – review and editing (equal). **Marek Słupek:** investigation (equal), resources (equal), writing – review and editing (equal). **Michał Chiliński:** visualization (equal), writing – review and editing (equal). **Krzysztof Damaziak:** conceptualization (lead), data curation (equal), investigation (lead), methodology (equal), writing – original draft (equal).

## Funding

The authors have nothing to report.

## Conflicts of Interest

The authors declare no conflicts of interest.

## Supporting information


**Figure S1:** Boxplots showing variation in mean egg traits (length, width, shell weight, shape index, diameter, surface area, degree of sphericity and volume) among shrike species: red‐backed shrike, woodchat shrike, lesser grey shrike and great grey shrike. Each box represents the interquartile range (IQR) with the median indicated by a horizontal line, whiskers extending to 1.5× IQR, and outliers shown as individual points.
**Figure S2:** Among‐year variation in egg morphology traits for four shrike species, red‐backed shrike, woodchat shrike, lesser grey shrike and great grey shrike, based on historical museum collections spanning 1888–1973. Each panel presents non‐parametric comparisons (Kruskal–Wallis test followed by Dunn's pairwise post hoc tests) for key reproductive traits, including egg length, width, shell weight, shape index, volume and clutch size. Boxes show interquartile ranges with medians, whiskers indicate data spread, and letters denote statistically significant differences among years (*p* < 0.05). *p*‐values from Kruskal–Wallis tests are provided in each panel.
**Figure S3:** Geographic variation in egg morphology traits of four shrike species, red‐backed shrike, woodchat shrike, lesser grey shrike and great grey shrike, across countries represented in the historical egg collection. Each panel shows mean values (± variation) of key egg traits—including egg length, width, diameter, surface area, volume, shell weight, shape index, degree of sphericity and clutch size—plotted by country. Sample sizes (*n*) for each country are indicated below the *x*‐axis.
**Figure S4:** Correlation heatmaps showing relationships among clutch size and mean egg traits (length, width, shell weight, shape index, diameter, surface area, degree of sphericity and volume) across all studied species (ALL) and separately for red‐backed shrike, woodchat shrike, lesser grey shrike and great grey shrike. Colour gradients represent Pearson correlation coefficients ranging from −1 (negative correlation, blue) to +1 (positive correlation, red).
**Table S1:** Results of pairwise Wilcoxon rank‐sum tests comparing egg and clutch traits between years for each shrike species: red‐backed shrike, woodchat shrike, lesser grey shrike and great grey shrike. The table includes trait name, compared years, raw (*p*) and adjusted *p*‐values, sample sizes for each year, and significance levels.
**Table S2:** Results of pairwise Wilcoxon rank‐sum tests comparing egg and clutch traits between countries for each shrike species: red‐backed shrike, woodchat shrike, lesser grey shrike and great grey shrike. The table includes trait name, compared years, raw (*p*) and adjusted *p*‐values, sample sizes for each year, and significance levels.
**Table S3:** Pearson correlation coefficients (*r*) between clutch and egg traits calculated across all species and separately for each shrike species: red‐backed shrike, woodchat shrike, lesser grey shrike and great grey shrike. The table lists pairs of variables, sample size (*n*), correlation coefficient (*r*), raw *p*‐values and Benjamini–Hochberg adjusted *p*‐values.

## Data Availability

The data that support the findings of this study are openly available in Figshare at https://doi.org/10.6084/m9.figshare.31950300.
