# Supplementary figures and images for: Egg Morphology and Clutch Size in Shrikes (Laniidae) From Historical Oological Collections: Interspecific Patterns and Modest Temporal Change
Source: Ecol Evol. 2026 Jul 20;16(7):e74065. doi: 10.1002/ece3.74065 (PMC13384774; doi:10.1002/ece3.74065)

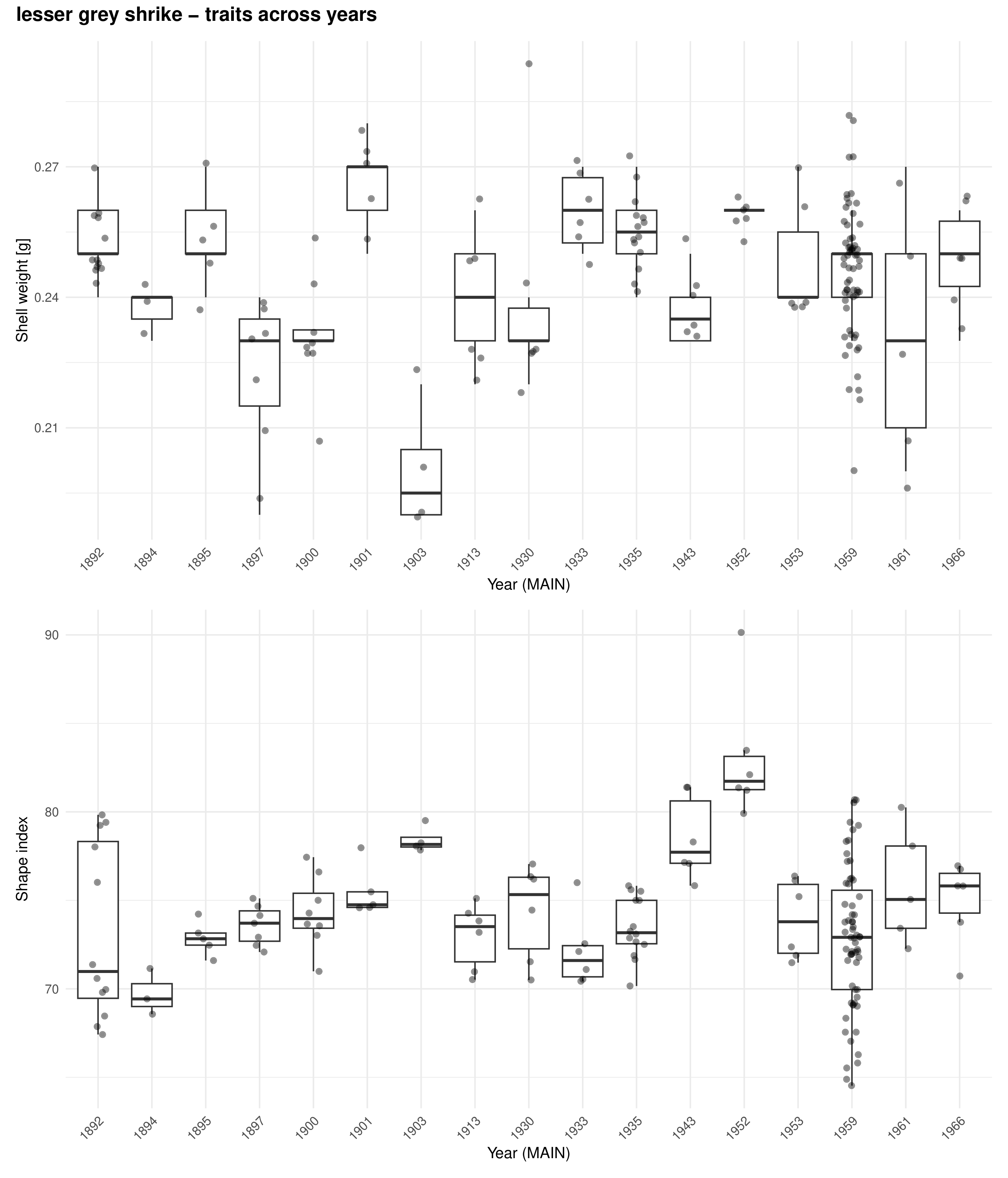

Supplement: Supplementary file 1 — Figure S1: Boxplots showing variation in mean egg traits (length, width, shell weight, shape index, diameter, surface area, degree of sphericity and volume) among shrike species: red‐backed shrike, woodchat shrike, lesser grey shrike and great grey shrike. Each box represents the interquartile range (IQR) with the median indicated by a horizontal line, whiskers extending to 1.5× IQR, and outliers shown as individual points. Figure S2: Among‐year variation in egg morphology traits for four shrike species, red‐backed shrike, woodchat shrike, lesser grey shrike and great grey shrike, based on historical museum collections spanning 1888–1973. Each panel presents non‐parametric comparisons (Kruskal–Wallis test followed by Dunn's pairwise post hoc tests) for key reproductive traits, including egg length, width, shell weight, shape index, volume and clutch size. Boxes show interquartile ranges with medians, whiskers indicate data spread, and letters denote statistically significant differences among years (p < 0.05). p‐values from Kruskal–Wallis tests are provided in each panel. Figure S3: Geographic variation in egg morphology traits of four shrike species, red‐backed shrike, woodchat shrike, lesser grey shrike and great grey shrike, across countries represented in the historical egg collection. Each panel shows mean values (± variation) of key egg traits—including egg length, width, diameter, surface area, volume, shell weight, shape index, degree of sphericity and clutch size—plotted by country. Sample sizes (n) for each country are indicated below the x‐axis. Figure S4: Correlation heatmaps showing relationships among clutch size and mean egg traits (length, width, shell weight, shape index, diameter, surface area, degree of sphericity and volume) across all studied species (ALL) and separately for red‐backed shrike, woodchat shrike, lesser grey shrike and great grey shrike. Colour gradients represent Pearson correlation coefficients ranging from −1 (negative correlati [file ECE3-16-e74065-s001.zip › Figure_S2j.png]

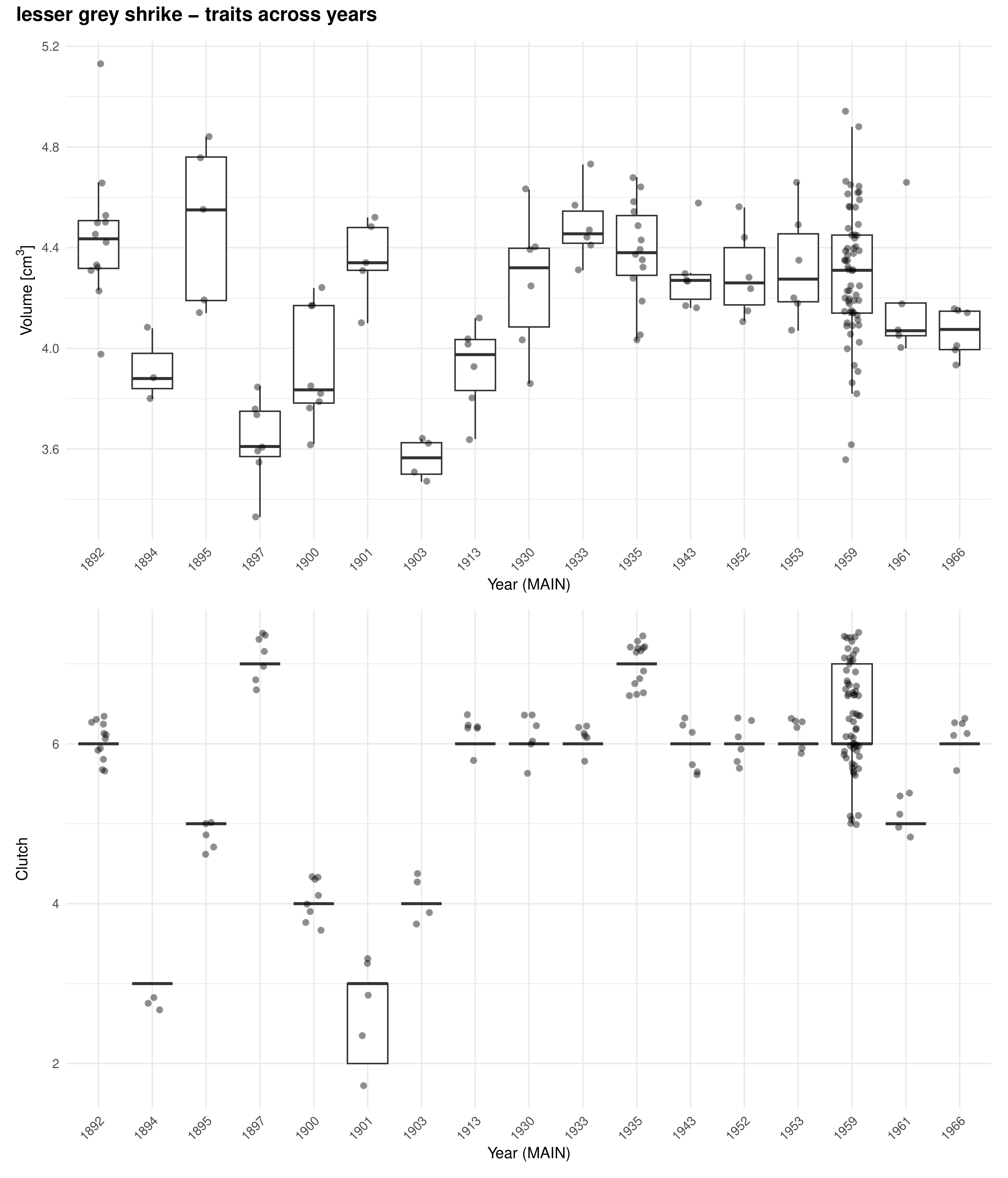

Supplement: Supplementary file 1 — Figure S1: Boxplots showing variation in mean egg traits (length, width, shell weight, shape index, diameter, surface area, degree of sphericity and volume) among shrike species: red‐backed shrike, woodchat shrike, lesser grey shrike and great grey shrike. Each box represents the interquartile range (IQR) with the median indicated by a horizontal line, whiskers extending to 1.5× IQR, and outliers shown as individual points. Figure S2: Among‐year variation in egg morphology traits for four shrike species, red‐backed shrike, woodchat shrike, lesser grey shrike and great grey shrike, based on historical museum collections spanning 1888–1973. Each panel presents non‐parametric comparisons (Kruskal–Wallis test followed by Dunn's pairwise post hoc tests) for key reproductive traits, including egg length, width, shell weight, shape index, volume and clutch size. Boxes show interquartile ranges with medians, whiskers indicate data spread, and letters denote statistically significant differences among years (p < 0.05). p‐values from Kruskal–Wallis tests are provided in each panel. Figure S3: Geographic variation in egg morphology traits of four shrike species, red‐backed shrike, woodchat shrike, lesser grey shrike and great grey shrike, across countries represented in the historical egg collection. Each panel shows mean values (± variation) of key egg traits—including egg length, width, diameter, surface area, volume, shell weight, shape index, degree of sphericity and clutch size—plotted by country. Sample sizes (n) for each country are indicated below the x‐axis. Figure S4: Correlation heatmaps showing relationships among clutch size and mean egg traits (length, width, shell weight, shape index, diameter, surface area, degree of sphericity and volume) across all studied species (ALL) and separately for red‐backed shrike, woodchat shrike, lesser grey shrike and great grey shrike. Colour gradients represent Pearson correlation coefficients ranging from −1 (negative correlati [file ECE3-16-e74065-s001.zip › Figure_S2k.png]

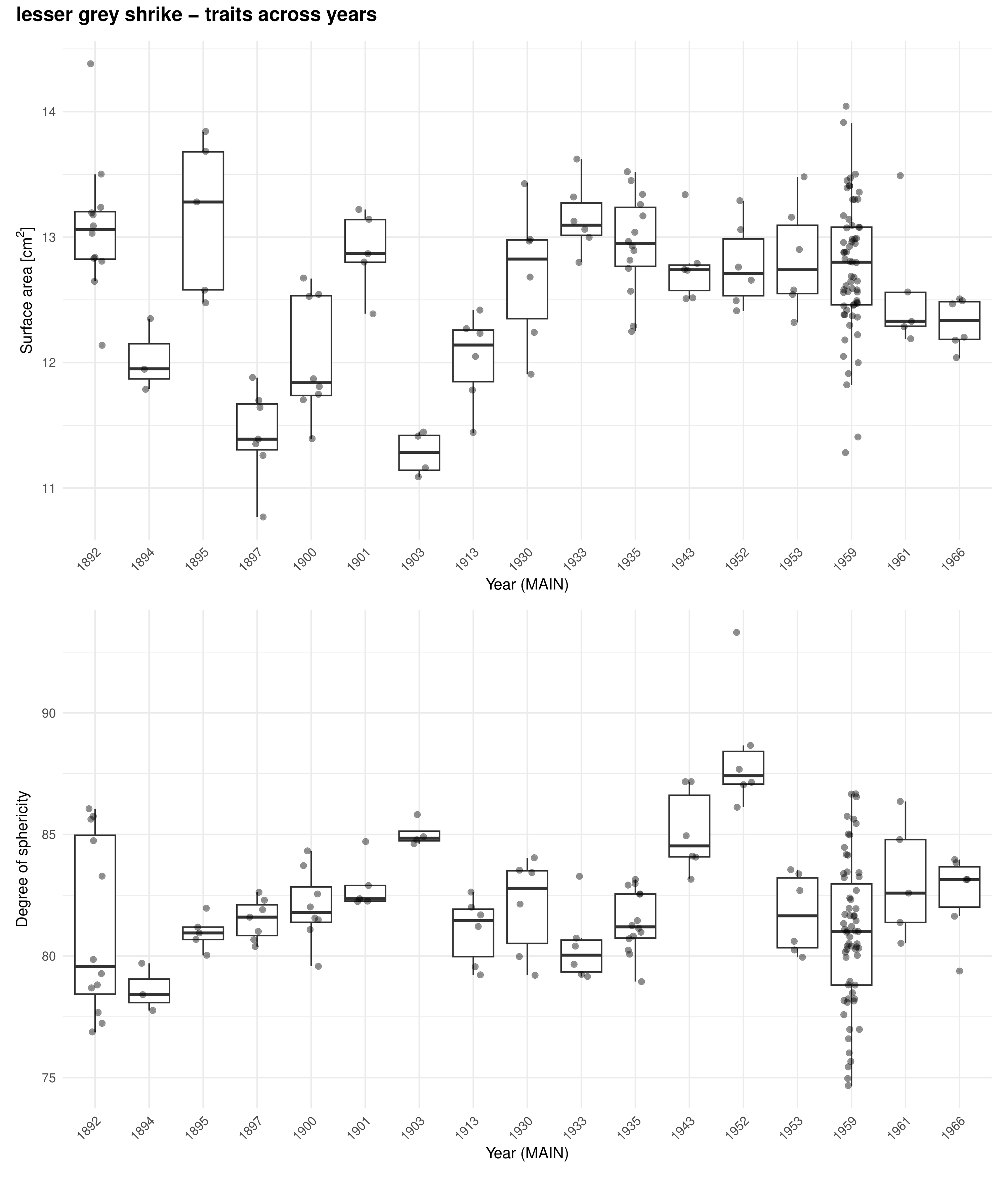

Supplement: Supplementary file 1 — Figure S1: Boxplots showing variation in mean egg traits (length, width, shell weight, shape index, diameter, surface area, degree of sphericity and volume) among shrike species: red‐backed shrike, woodchat shrike, lesser grey shrike and great grey shrike. Each box represents the interquartile range (IQR) with the median indicated by a horizontal line, whiskers extending to 1.5× IQR, and outliers shown as individual points. Figure S2: Among‐year variation in egg morphology traits for four shrike species, red‐backed shrike, woodchat shrike, lesser grey shrike and great grey shrike, based on historical museum collections spanning 1888–1973. Each panel presents non‐parametric comparisons (Kruskal–Wallis test followed by Dunn's pairwise post hoc tests) for key reproductive traits, including egg length, width, shell weight, shape index, volume and clutch size. Boxes show interquartile ranges with medians, whiskers indicate data spread, and letters denote statistically significant differences among years (p < 0.05). p‐values from Kruskal–Wallis tests are provided in each panel. Figure S3: Geographic variation in egg morphology traits of four shrike species, red‐backed shrike, woodchat shrike, lesser grey shrike and great grey shrike, across countries represented in the historical egg collection. Each panel shows mean values (± variation) of key egg traits—including egg length, width, diameter, surface area, volume, shell weight, shape index, degree of sphericity and clutch size—plotted by country. Sample sizes (n) for each country are indicated below the x‐axis. Figure S4: Correlation heatmaps showing relationships among clutch size and mean egg traits (length, width, shell weight, shape index, diameter, surface area, degree of sphericity and volume) across all studied species (ALL) and separately for red‐backed shrike, woodchat shrike, lesser grey shrike and great grey shrike. Colour gradients represent Pearson correlation coefficients ranging from −1 (negative correlati [file ECE3-16-e74065-s001.zip › Figure_S2l.png]

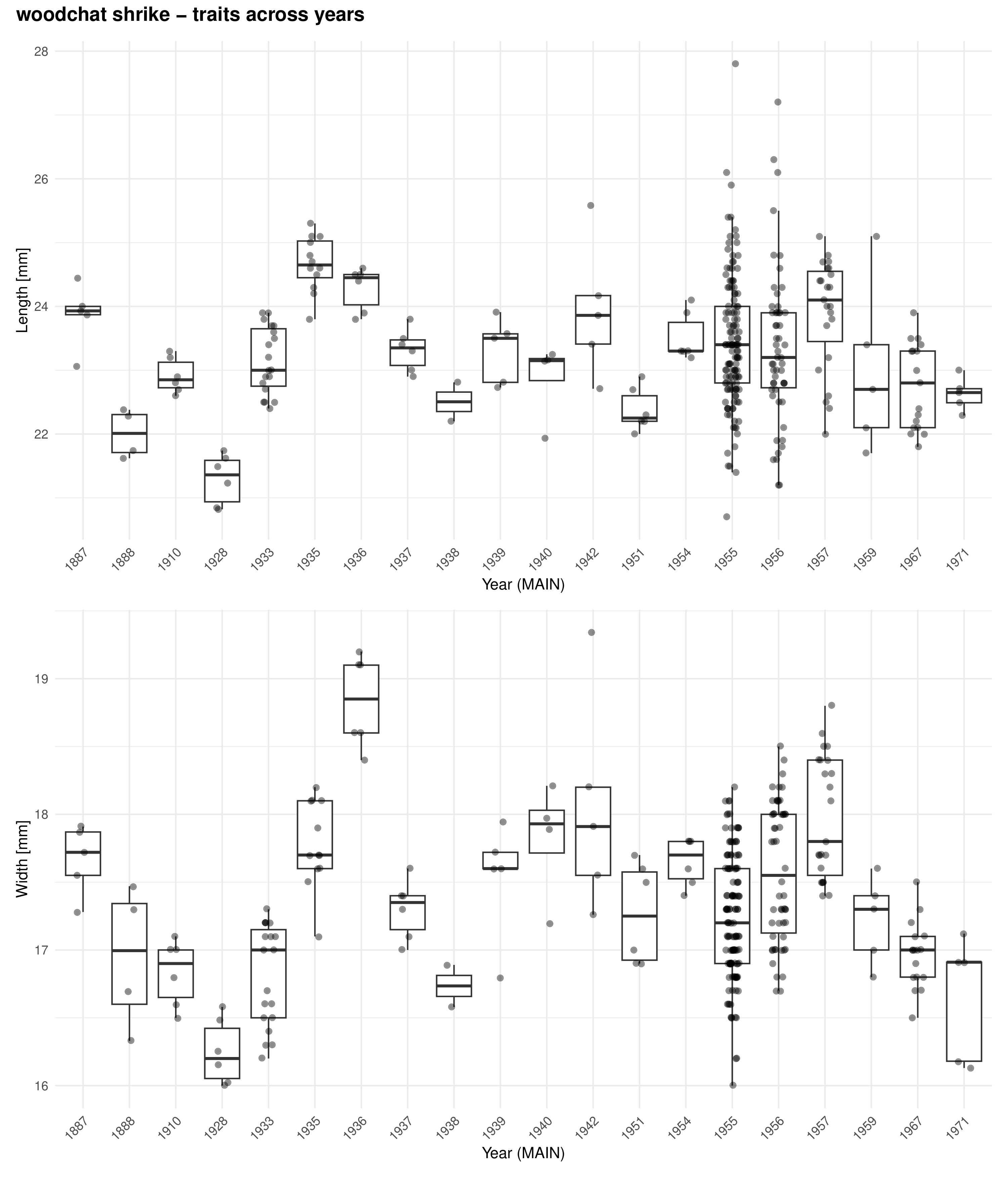

Supplement: Supplementary file 1 — Figure S1: Boxplots showing variation in mean egg traits (length, width, shell weight, shape index, diameter, surface area, degree of sphericity and volume) among shrike species: red‐backed shrike, woodchat shrike, lesser grey shrike and great grey shrike. Each box represents the interquartile range (IQR) with the median indicated by a horizontal line, whiskers extending to 1.5× IQR, and outliers shown as individual points. Figure S2: Among‐year variation in egg morphology traits for four shrike species, red‐backed shrike, woodchat shrike, lesser grey shrike and great grey shrike, based on historical museum collections spanning 1888–1973. Each panel presents non‐parametric comparisons (Kruskal–Wallis test followed by Dunn's pairwise post hoc tests) for key reproductive traits, including egg length, width, shell weight, shape index, volume and clutch size. Boxes show interquartile ranges with medians, whiskers indicate data spread, and letters denote statistically significant differences among years (p < 0.05). p‐values from Kruskal–Wallis tests are provided in each panel. Figure S3: Geographic variation in egg morphology traits of four shrike species, red‐backed shrike, woodchat shrike, lesser grey shrike and great grey shrike, across countries represented in the historical egg collection. Each panel shows mean values (± variation) of key egg traits—including egg length, width, diameter, surface area, volume, shell weight, shape index, degree of sphericity and clutch size—plotted by country. Sample sizes (n) for each country are indicated below the x‐axis. Figure S4: Correlation heatmaps showing relationships among clutch size and mean egg traits (length, width, shell weight, shape index, diameter, surface area, degree of sphericity and volume) across all studied species (ALL) and separately for red‐backed shrike, woodchat shrike, lesser grey shrike and great grey shrike. Colour gradients represent Pearson correlation coefficients ranging from −1 (negative correlati [file ECE3-16-e74065-s001.zip › Figure_S2m.png]

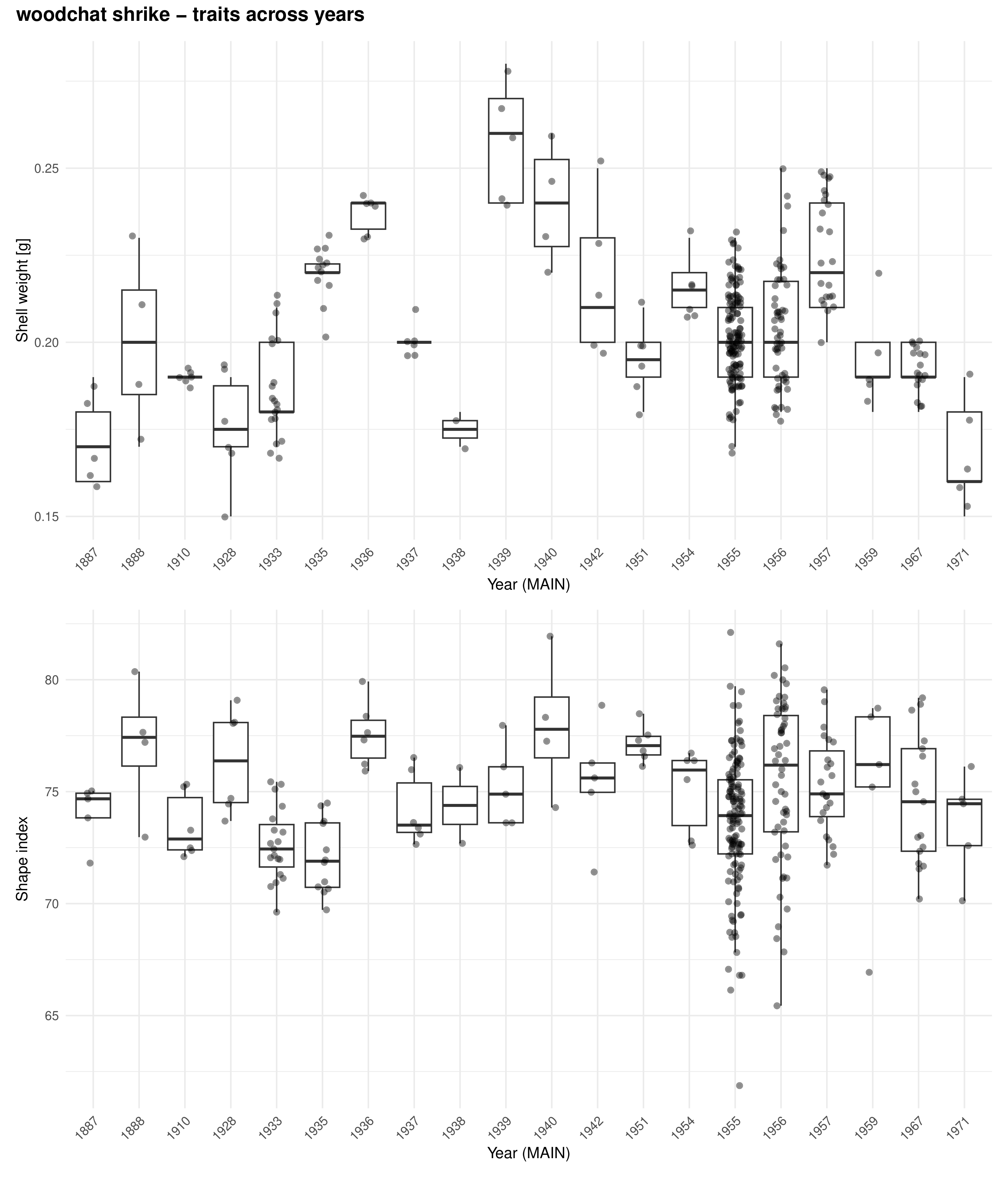

Supplement: Supplementary file 1 — Figure S1: Boxplots showing variation in mean egg traits (length, width, shell weight, shape index, diameter, surface area, degree of sphericity and volume) among shrike species: red‐backed shrike, woodchat shrike, lesser grey shrike and great grey shrike. Each box represents the interquartile range (IQR) with the median indicated by a horizontal line, whiskers extending to 1.5× IQR, and outliers shown as individual points. Figure S2: Among‐year variation in egg morphology traits for four shrike species, red‐backed shrike, woodchat shrike, lesser grey shrike and great grey shrike, based on historical museum collections spanning 1888–1973. Each panel presents non‐parametric comparisons (Kruskal–Wallis test followed by Dunn's pairwise post hoc tests) for key reproductive traits, including egg length, width, shell weight, shape index, volume and clutch size. Boxes show interquartile ranges with medians, whiskers indicate data spread, and letters denote statistically significant differences among years (p < 0.05). p‐values from Kruskal–Wallis tests are provided in each panel. Figure S3: Geographic variation in egg morphology traits of four shrike species, red‐backed shrike, woodchat shrike, lesser grey shrike and great grey shrike, across countries represented in the historical egg collection. Each panel shows mean values (± variation) of key egg traits—including egg length, width, diameter, surface area, volume, shell weight, shape index, degree of sphericity and clutch size—plotted by country. Sample sizes (n) for each country are indicated below the x‐axis. Figure S4: Correlation heatmaps showing relationships among clutch size and mean egg traits (length, width, shell weight, shape index, diameter, surface area, degree of sphericity and volume) across all studied species (ALL) and separately for red‐backed shrike, woodchat shrike, lesser grey shrike and great grey shrike. Colour gradients represent Pearson correlation coefficients ranging from −1 (negative correlati [file ECE3-16-e74065-s001.zip › Figure_S2n.png]

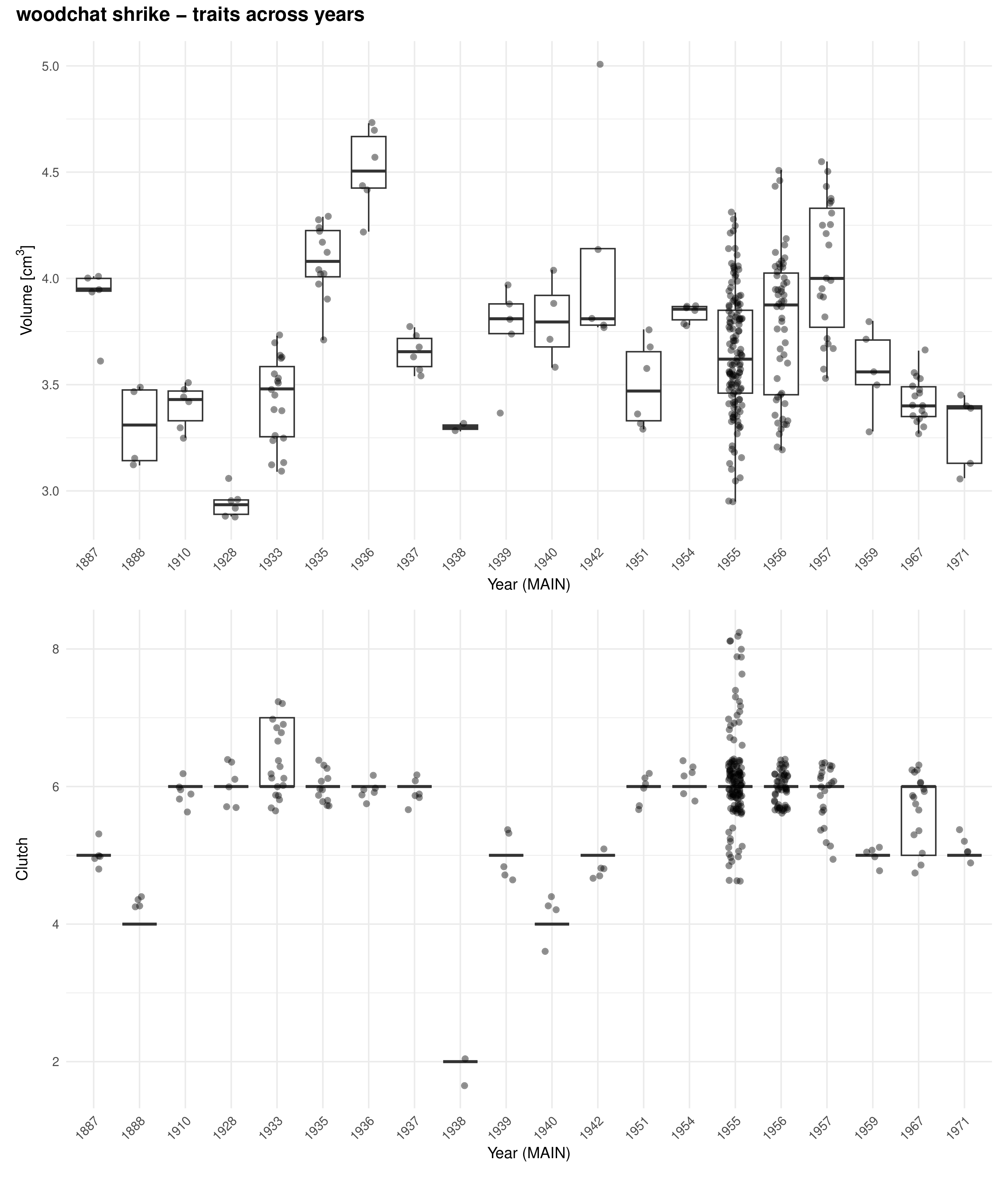

Supplement: Supplementary file 1 — Figure S1: Boxplots showing variation in mean egg traits (length, width, shell weight, shape index, diameter, surface area, degree of sphericity and volume) among shrike species: red‐backed shrike, woodchat shrike, lesser grey shrike and great grey shrike. Each box represents the interquartile range (IQR) with the median indicated by a horizontal line, whiskers extending to 1.5× IQR, and outliers shown as individual points. Figure S2: Among‐year variation in egg morphology traits for four shrike species, red‐backed shrike, woodchat shrike, lesser grey shrike and great grey shrike, based on historical museum collections spanning 1888–1973. Each panel presents non‐parametric comparisons (Kruskal–Wallis test followed by Dunn's pairwise post hoc tests) for key reproductive traits, including egg length, width, shell weight, shape index, volume and clutch size. Boxes show interquartile ranges with medians, whiskers indicate data spread, and letters denote statistically significant differences among years (p < 0.05). p‐values from Kruskal–Wallis tests are provided in each panel. Figure S3: Geographic variation in egg morphology traits of four shrike species, red‐backed shrike, woodchat shrike, lesser grey shrike and great grey shrike, across countries represented in the historical egg collection. Each panel shows mean values (± variation) of key egg traits—including egg length, width, diameter, surface area, volume, shell weight, shape index, degree of sphericity and clutch size—plotted by country. Sample sizes (n) for each country are indicated below the x‐axis. Figure S4: Correlation heatmaps showing relationships among clutch size and mean egg traits (length, width, shell weight, shape index, diameter, surface area, degree of sphericity and volume) across all studied species (ALL) and separately for red‐backed shrike, woodchat shrike, lesser grey shrike and great grey shrike. Colour gradients represent Pearson correlation coefficients ranging from −1 (negative correlati [file ECE3-16-e74065-s001.zip › Figure_S2o.png]

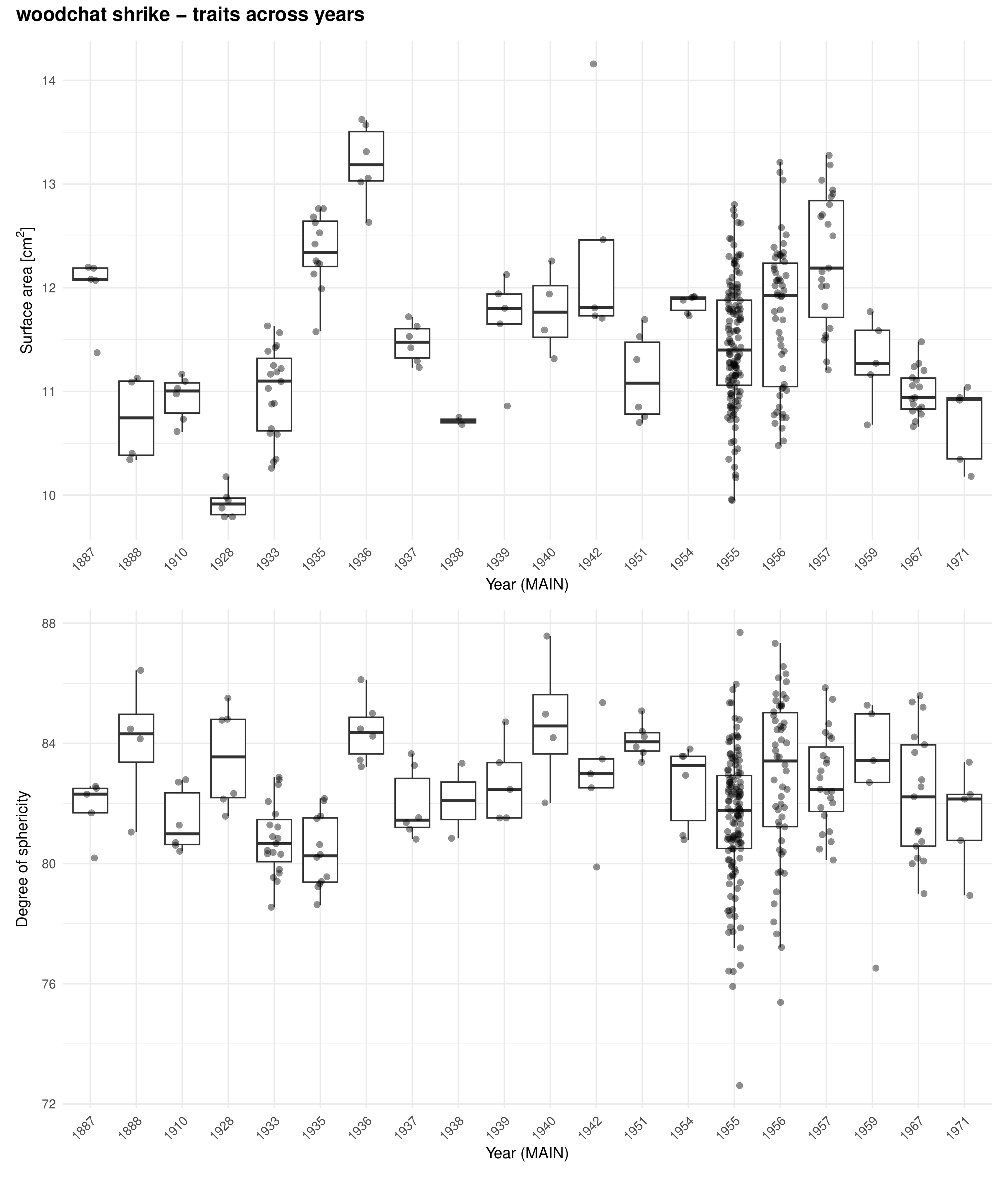

Supplement: Supplementary file 1 — Figure S1: Boxplots showing variation in mean egg traits (length, width, shell weight, shape index, diameter, surface area, degree of sphericity and volume) among shrike species: red‐backed shrike, woodchat shrike, lesser grey shrike and great grey shrike. Each box represents the interquartile range (IQR) with the median indicated by a horizontal line, whiskers extending to 1.5× IQR, and outliers shown as individual points. Figure S2: Among‐year variation in egg morphology traits for four shrike species, red‐backed shrike, woodchat shrike, lesser grey shrike and great grey shrike, based on historical museum collections spanning 1888–1973. Each panel presents non‐parametric comparisons (Kruskal–Wallis test followed by Dunn's pairwise post hoc tests) for key reproductive traits, including egg length, width, shell weight, shape index, volume and clutch size. Boxes show interquartile ranges with medians, whiskers indicate data spread, and letters denote statistically significant differences among years (p < 0.05). p‐values from Kruskal–Wallis tests are provided in each panel. Figure S3: Geographic variation in egg morphology traits of four shrike species, red‐backed shrike, woodchat shrike, lesser grey shrike and great grey shrike, across countries represented in the historical egg collection. Each panel shows mean values (± variation) of key egg traits—including egg length, width, diameter, surface area, volume, shell weight, shape index, degree of sphericity and clutch size—plotted by country. Sample sizes (n) for each country are indicated below the x‐axis. Figure S4: Correlation heatmaps showing relationships among clutch size and mean egg traits (length, width, shell weight, shape index, diameter, surface area, degree of sphericity and volume) across all studied species (ALL) and separately for red‐backed shrike, woodchat shrike, lesser grey shrike and great grey shrike. Colour gradients represent Pearson correlation coefficients ranging from −1 (negative correlati [file ECE3-16-e74065-s001.zip › Figure_S2p.png]

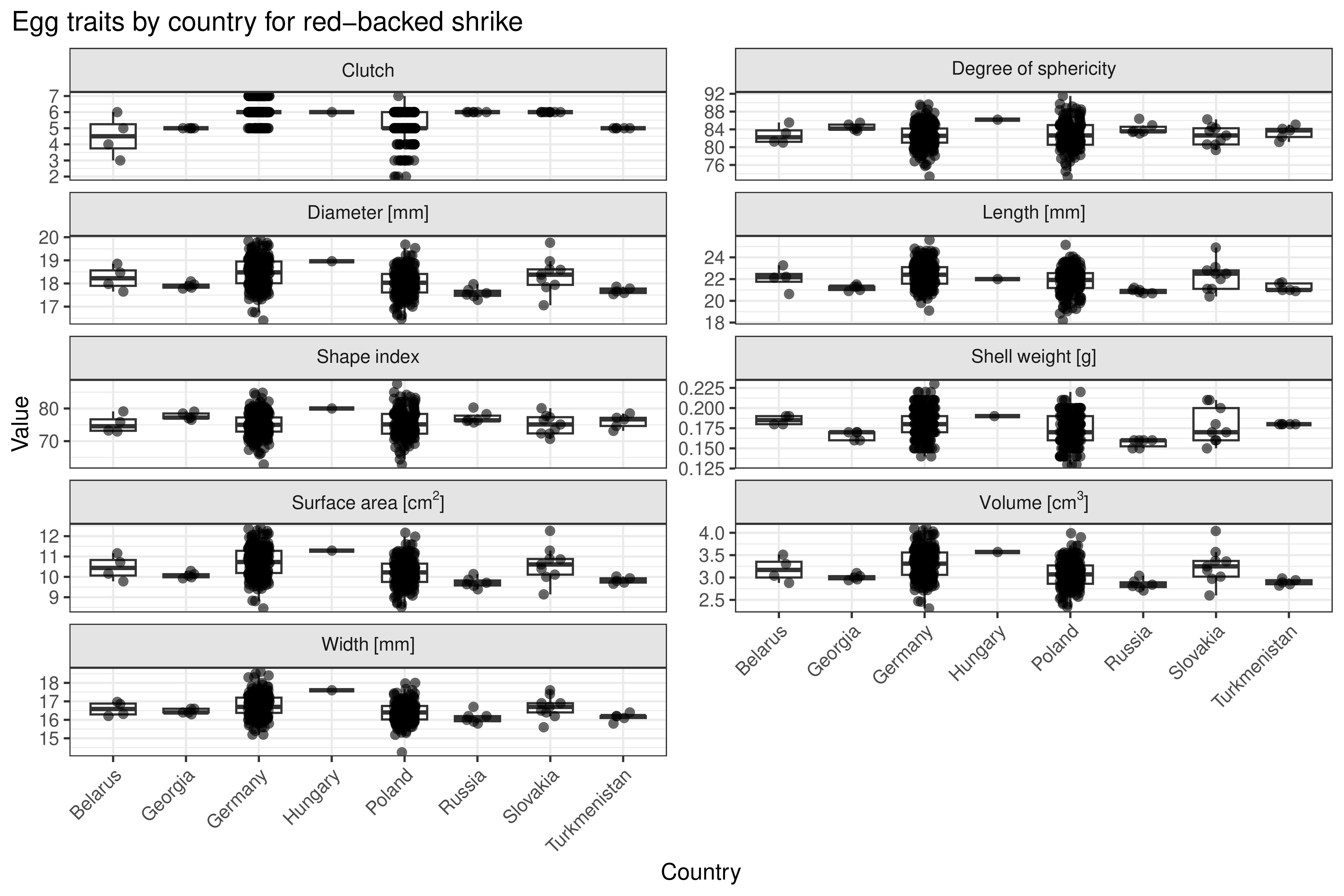

Supplement: Supplementary file 1 — Figure S1: Boxplots showing variation in mean egg traits (length, width, shell weight, shape index, diameter, surface area, degree of sphericity and volume) among shrike species: red‐backed shrike, woodchat shrike, lesser grey shrike and great grey shrike. Each box represents the interquartile range (IQR) with the median indicated by a horizontal line, whiskers extending to 1.5× IQR, and outliers shown as individual points. Figure S2: Among‐year variation in egg morphology traits for four shrike species, red‐backed shrike, woodchat shrike, lesser grey shrike and great grey shrike, based on historical museum collections spanning 1888–1973. Each panel presents non‐parametric comparisons (Kruskal–Wallis test followed by Dunn's pairwise post hoc tests) for key reproductive traits, including egg length, width, shell weight, shape index, volume and clutch size. Boxes show interquartile ranges with medians, whiskers indicate data spread, and letters denote statistically significant differences among years (p < 0.05). p‐values from Kruskal–Wallis tests are provided in each panel. Figure S3: Geographic variation in egg morphology traits of four shrike species, red‐backed shrike, woodchat shrike, lesser grey shrike and great grey shrike, across countries represented in the historical egg collection. Each panel shows mean values (± variation) of key egg traits—including egg length, width, diameter, surface area, volume, shell weight, shape index, degree of sphericity and clutch size—plotted by country. Sample sizes (n) for each country are indicated below the x‐axis. Figure S4: Correlation heatmaps showing relationships among clutch size and mean egg traits (length, width, shell weight, shape index, diameter, surface area, degree of sphericity and volume) across all studied species (ALL) and separately for red‐backed shrike, woodchat shrike, lesser grey shrike and great grey shrike. Colour gradients represent Pearson correlation coefficients ranging from −1 (negative correlati [file ECE3-16-e74065-s001.zip › Figure_S3a.png]

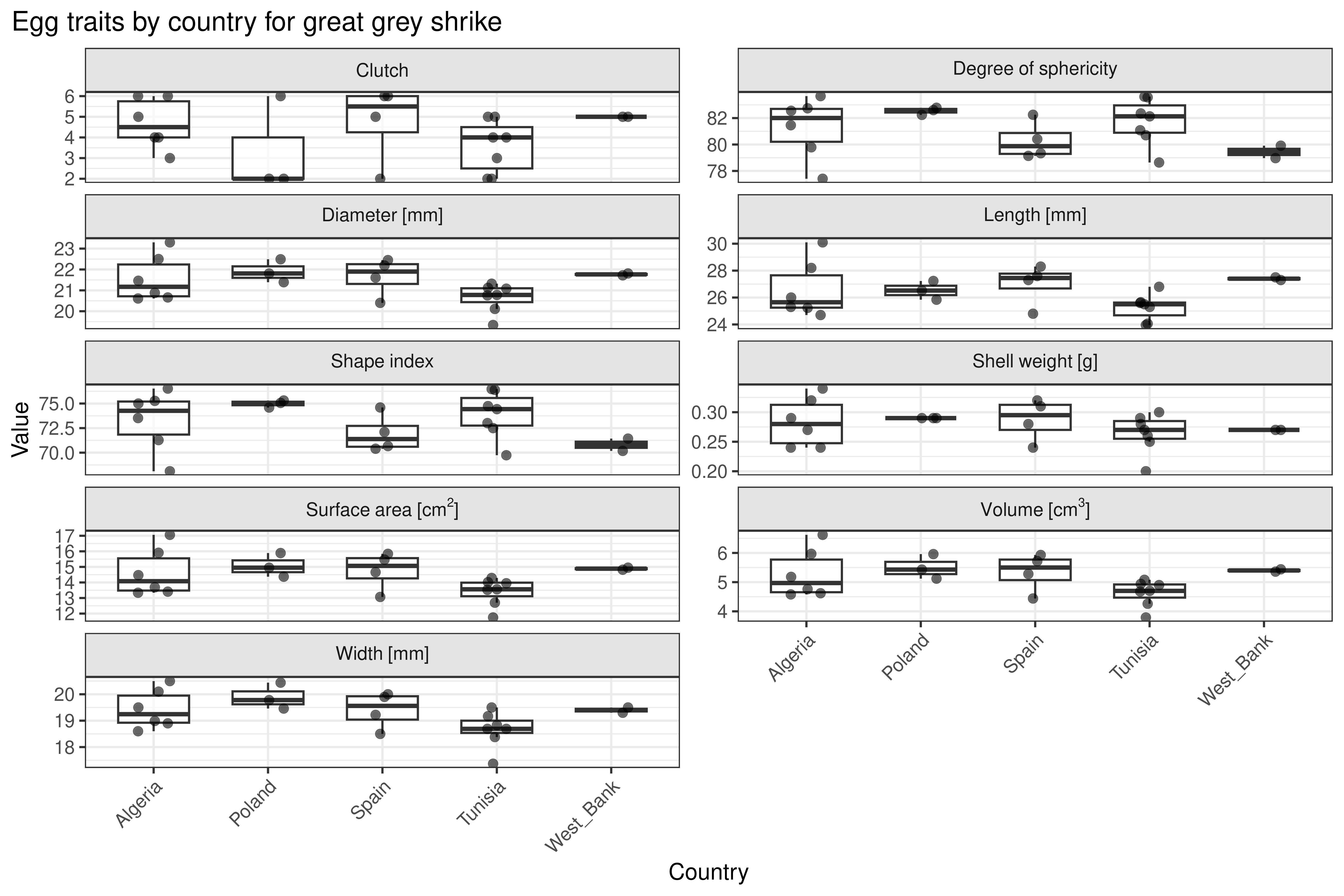

Supplement: Supplementary file 1 — Figure S1: Boxplots showing variation in mean egg traits (length, width, shell weight, shape index, diameter, surface area, degree of sphericity and volume) among shrike species: red‐backed shrike, woodchat shrike, lesser grey shrike and great grey shrike. Each box represents the interquartile range (IQR) with the median indicated by a horizontal line, whiskers extending to 1.5× IQR, and outliers shown as individual points. Figure S2: Among‐year variation in egg morphology traits for four shrike species, red‐backed shrike, woodchat shrike, lesser grey shrike and great grey shrike, based on historical museum collections spanning 1888–1973. Each panel presents non‐parametric comparisons (Kruskal–Wallis test followed by Dunn's pairwise post hoc tests) for key reproductive traits, including egg length, width, shell weight, shape index, volume and clutch size. Boxes show interquartile ranges with medians, whiskers indicate data spread, and letters denote statistically significant differences among years (p < 0.05). p‐values from Kruskal–Wallis tests are provided in each panel. Figure S3: Geographic variation in egg morphology traits of four shrike species, red‐backed shrike, woodchat shrike, lesser grey shrike and great grey shrike, across countries represented in the historical egg collection. Each panel shows mean values (± variation) of key egg traits—including egg length, width, diameter, surface area, volume, shell weight, shape index, degree of sphericity and clutch size—plotted by country. Sample sizes (n) for each country are indicated below the x‐axis. Figure S4: Correlation heatmaps showing relationships among clutch size and mean egg traits (length, width, shell weight, shape index, diameter, surface area, degree of sphericity and volume) across all studied species (ALL) and separately for red‐backed shrike, woodchat shrike, lesser grey shrike and great grey shrike. Colour gradients represent Pearson correlation coefficients ranging from −1 (negative correlati [file ECE3-16-e74065-s001.zip › Figure_S3b.png]

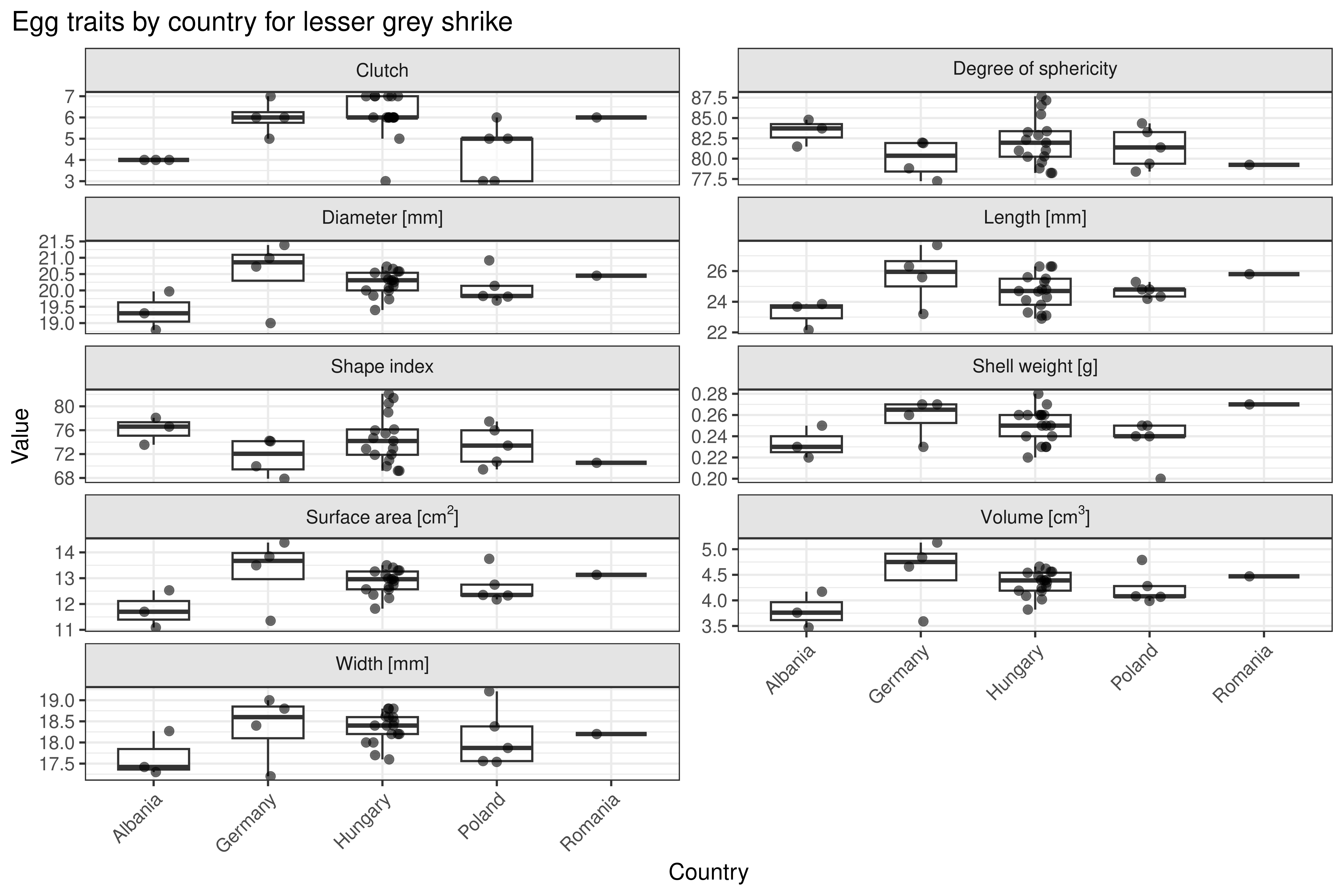

Supplement: Supplementary file 1 — Figure S1: Boxplots showing variation in mean egg traits (length, width, shell weight, shape index, diameter, surface area, degree of sphericity and volume) among shrike species: red‐backed shrike, woodchat shrike, lesser grey shrike and great grey shrike. Each box represents the interquartile range (IQR) with the median indicated by a horizontal line, whiskers extending to 1.5× IQR, and outliers shown as individual points. Figure S2: Among‐year variation in egg morphology traits for four shrike species, red‐backed shrike, woodchat shrike, lesser grey shrike and great grey shrike, based on historical museum collections spanning 1888–1973. Each panel presents non‐parametric comparisons (Kruskal–Wallis test followed by Dunn's pairwise post hoc tests) for key reproductive traits, including egg length, width, shell weight, shape index, volume and clutch size. Boxes show interquartile ranges with medians, whiskers indicate data spread, and letters denote statistically significant differences among years (p < 0.05). p‐values from Kruskal–Wallis tests are provided in each panel. Figure S3: Geographic variation in egg morphology traits of four shrike species, red‐backed shrike, woodchat shrike, lesser grey shrike and great grey shrike, across countries represented in the historical egg collection. Each panel shows mean values (± variation) of key egg traits—including egg length, width, diameter, surface area, volume, shell weight, shape index, degree of sphericity and clutch size—plotted by country. Sample sizes (n) for each country are indicated below the x‐axis. Figure S4: Correlation heatmaps showing relationships among clutch size and mean egg traits (length, width, shell weight, shape index, diameter, surface area, degree of sphericity and volume) across all studied species (ALL) and separately for red‐backed shrike, woodchat shrike, lesser grey shrike and great grey shrike. Colour gradients represent Pearson correlation coefficients ranging from −1 (negative correlati [file ECE3-16-e74065-s001.zip › Figure_S3c.png]

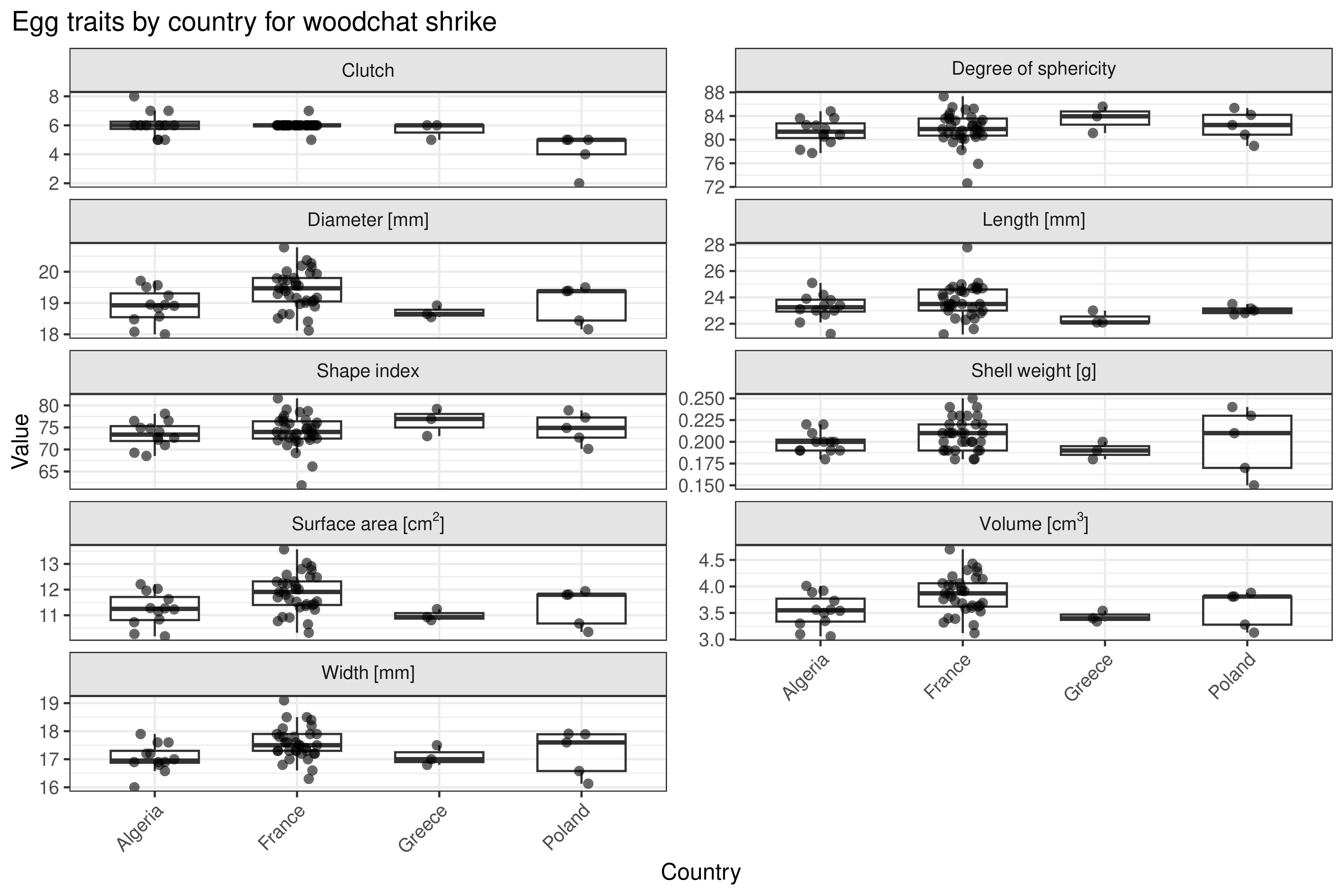

Supplement: Supplementary file 1 — Figure S1: Boxplots showing variation in mean egg traits (length, width, shell weight, shape index, diameter, surface area, degree of sphericity and volume) among shrike species: red‐backed shrike, woodchat shrike, lesser grey shrike and great grey shrike. Each box represents the interquartile range (IQR) with the median indicated by a horizontal line, whiskers extending to 1.5× IQR, and outliers shown as individual points. Figure S2: Among‐year variation in egg morphology traits for four shrike species, red‐backed shrike, woodchat shrike, lesser grey shrike and great grey shrike, based on historical museum collections spanning 1888–1973. Each panel presents non‐parametric comparisons (Kruskal–Wallis test followed by Dunn's pairwise post hoc tests) for key reproductive traits, including egg length, width, shell weight, shape index, volume and clutch size. Boxes show interquartile ranges with medians, whiskers indicate data spread, and letters denote statistically significant differences among years (p < 0.05). p‐values from Kruskal–Wallis tests are provided in each panel. Figure S3: Geographic variation in egg morphology traits of four shrike species, red‐backed shrike, woodchat shrike, lesser grey shrike and great grey shrike, across countries represented in the historical egg collection. Each panel shows mean values (± variation) of key egg traits—including egg length, width, diameter, surface area, volume, shell weight, shape index, degree of sphericity and clutch size—plotted by country. Sample sizes (n) for each country are indicated below the x‐axis. Figure S4: Correlation heatmaps showing relationships among clutch size and mean egg traits (length, width, shell weight, shape index, diameter, surface area, degree of sphericity and volume) across all studied species (ALL) and separately for red‐backed shrike, woodchat shrike, lesser grey shrike and great grey shrike. Colour gradients represent Pearson correlation coefficients ranging from −1 (negative correlati [file ECE3-16-e74065-s001.zip › Figure_S3d.png]

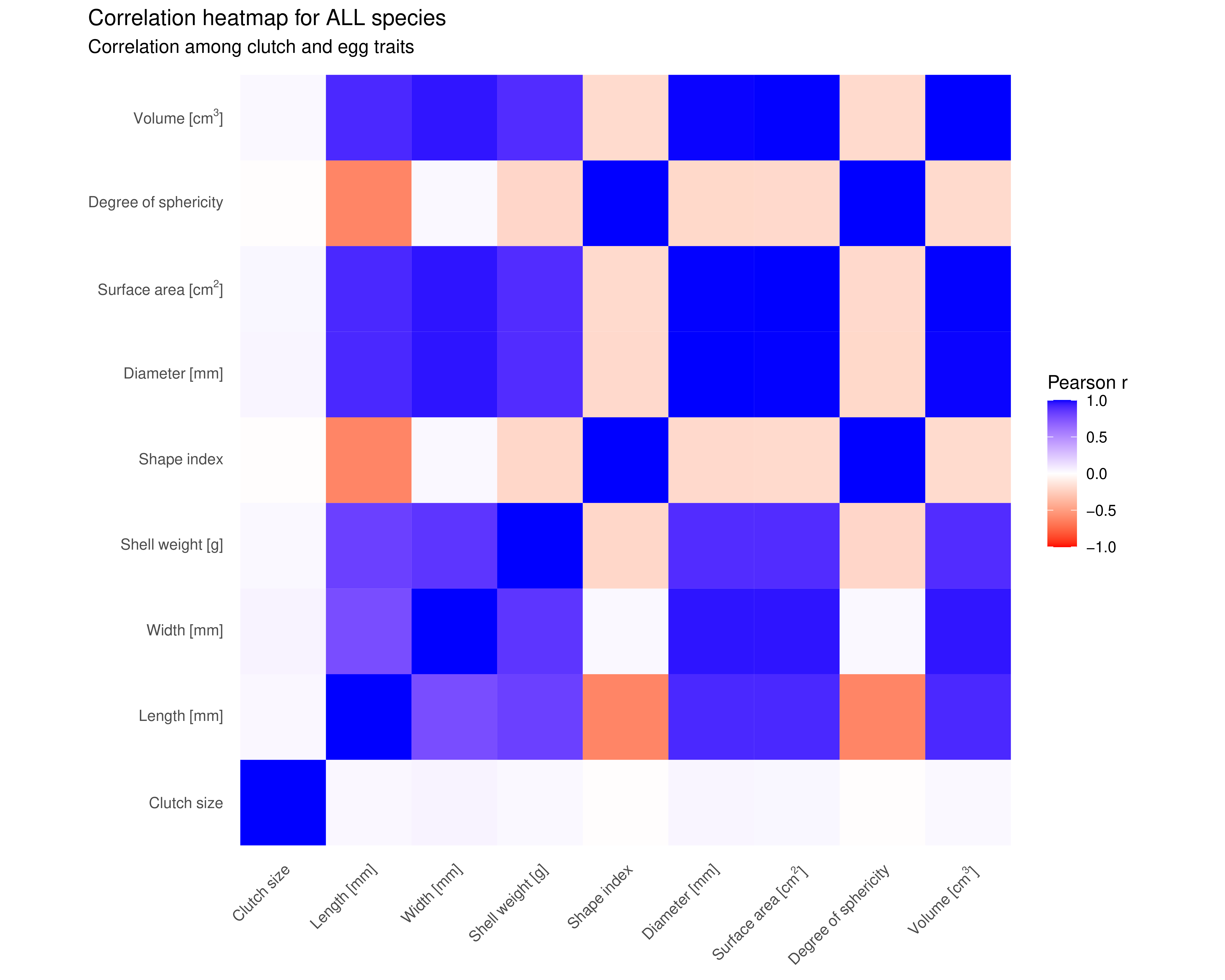

Supplement: Supplementary file 1 — Figure S1: Boxplots showing variation in mean egg traits (length, width, shell weight, shape index, diameter, surface area, degree of sphericity and volume) among shrike species: red‐backed shrike, woodchat shrike, lesser grey shrike and great grey shrike. Each box represents the interquartile range (IQR) with the median indicated by a horizontal line, whiskers extending to 1.5× IQR, and outliers shown as individual points. Figure S2: Among‐year variation in egg morphology traits for four shrike species, red‐backed shrike, woodchat shrike, lesser grey shrike and great grey shrike, based on historical museum collections spanning 1888–1973. Each panel presents non‐parametric comparisons (Kruskal–Wallis test followed by Dunn's pairwise post hoc tests) for key reproductive traits, including egg length, width, shell weight, shape index, volume and clutch size. Boxes show interquartile ranges with medians, whiskers indicate data spread, and letters denote statistically significant differences among years (p < 0.05). p‐values from Kruskal–Wallis tests are provided in each panel. Figure S3: Geographic variation in egg morphology traits of four shrike species, red‐backed shrike, woodchat shrike, lesser grey shrike and great grey shrike, across countries represented in the historical egg collection. Each panel shows mean values (± variation) of key egg traits—including egg length, width, diameter, surface area, volume, shell weight, shape index, degree of sphericity and clutch size—plotted by country. Sample sizes (n) for each country are indicated below the x‐axis. Figure S4: Correlation heatmaps showing relationships among clutch size and mean egg traits (length, width, shell weight, shape index, diameter, surface area, degree of sphericity and volume) across all studied species (ALL) and separately for red‐backed shrike, woodchat shrike, lesser grey shrike and great grey shrike. Colour gradients represent Pearson correlation coefficients ranging from −1 (negative correlati [file ECE3-16-e74065-s001.zip › Figure_S4a.png]

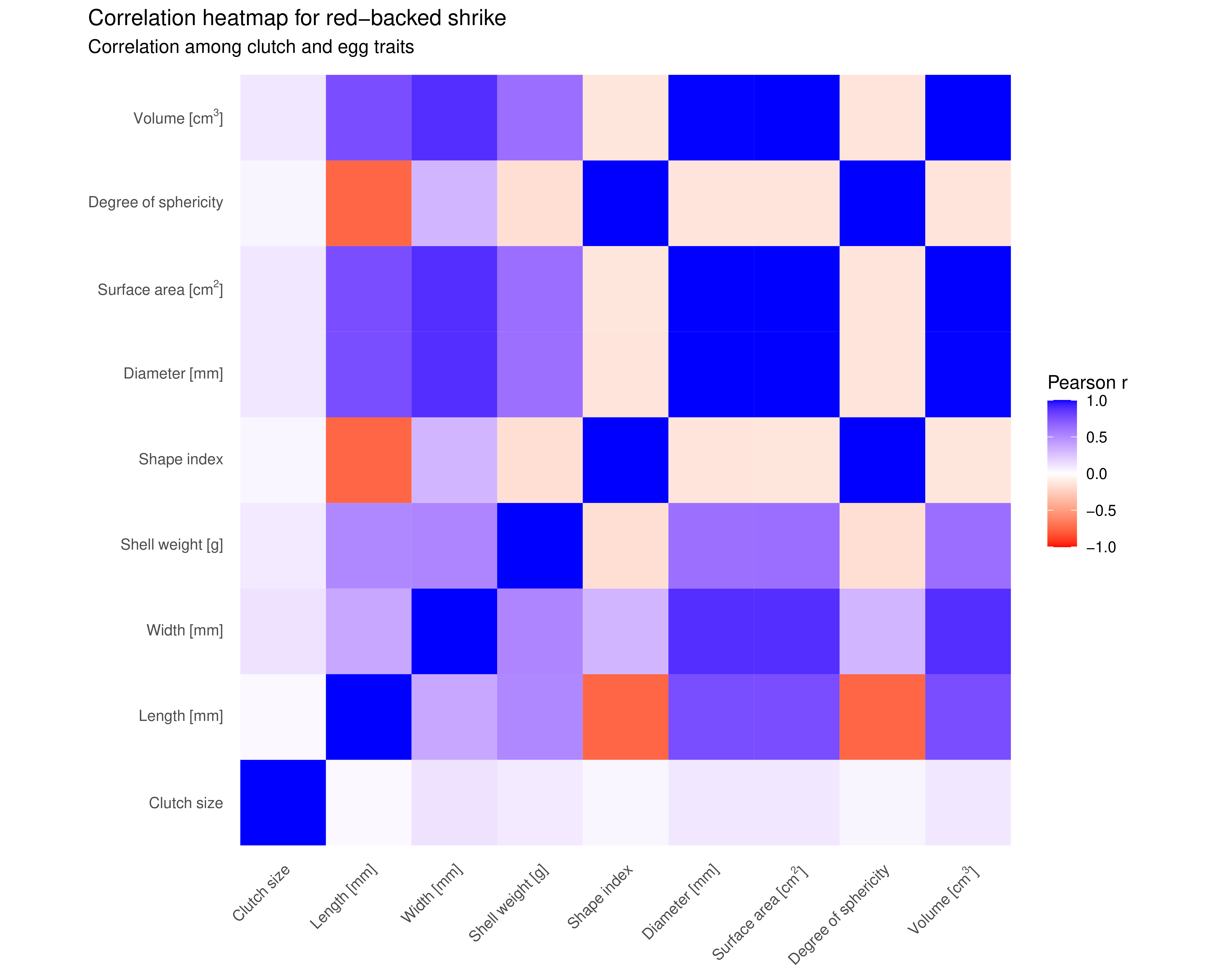

Supplement: Supplementary file 1 — Figure S1: Boxplots showing variation in mean egg traits (length, width, shell weight, shape index, diameter, surface area, degree of sphericity and volume) among shrike species: red‐backed shrike, woodchat shrike, lesser grey shrike and great grey shrike. Each box represents the interquartile range (IQR) with the median indicated by a horizontal line, whiskers extending to 1.5× IQR, and outliers shown as individual points. Figure S2: Among‐year variation in egg morphology traits for four shrike species, red‐backed shrike, woodchat shrike, lesser grey shrike and great grey shrike, based on historical museum collections spanning 1888–1973. Each panel presents non‐parametric comparisons (Kruskal–Wallis test followed by Dunn's pairwise post hoc tests) for key reproductive traits, including egg length, width, shell weight, shape index, volume and clutch size. Boxes show interquartile ranges with medians, whiskers indicate data spread, and letters denote statistically significant differences among years (p < 0.05). p‐values from Kruskal–Wallis tests are provided in each panel. Figure S3: Geographic variation in egg morphology traits of four shrike species, red‐backed shrike, woodchat shrike, lesser grey shrike and great grey shrike, across countries represented in the historical egg collection. Each panel shows mean values (± variation) of key egg traits—including egg length, width, diameter, surface area, volume, shell weight, shape index, degree of sphericity and clutch size—plotted by country. Sample sizes (n) for each country are indicated below the x‐axis. Figure S4: Correlation heatmaps showing relationships among clutch size and mean egg traits (length, width, shell weight, shape index, diameter, surface area, degree of sphericity and volume) across all studied species (ALL) and separately for red‐backed shrike, woodchat shrike, lesser grey shrike and great grey shrike. Colour gradients represent Pearson correlation coefficients ranging from −1 (negative correlati [file ECE3-16-e74065-s001.zip › Figure_S4b.png]

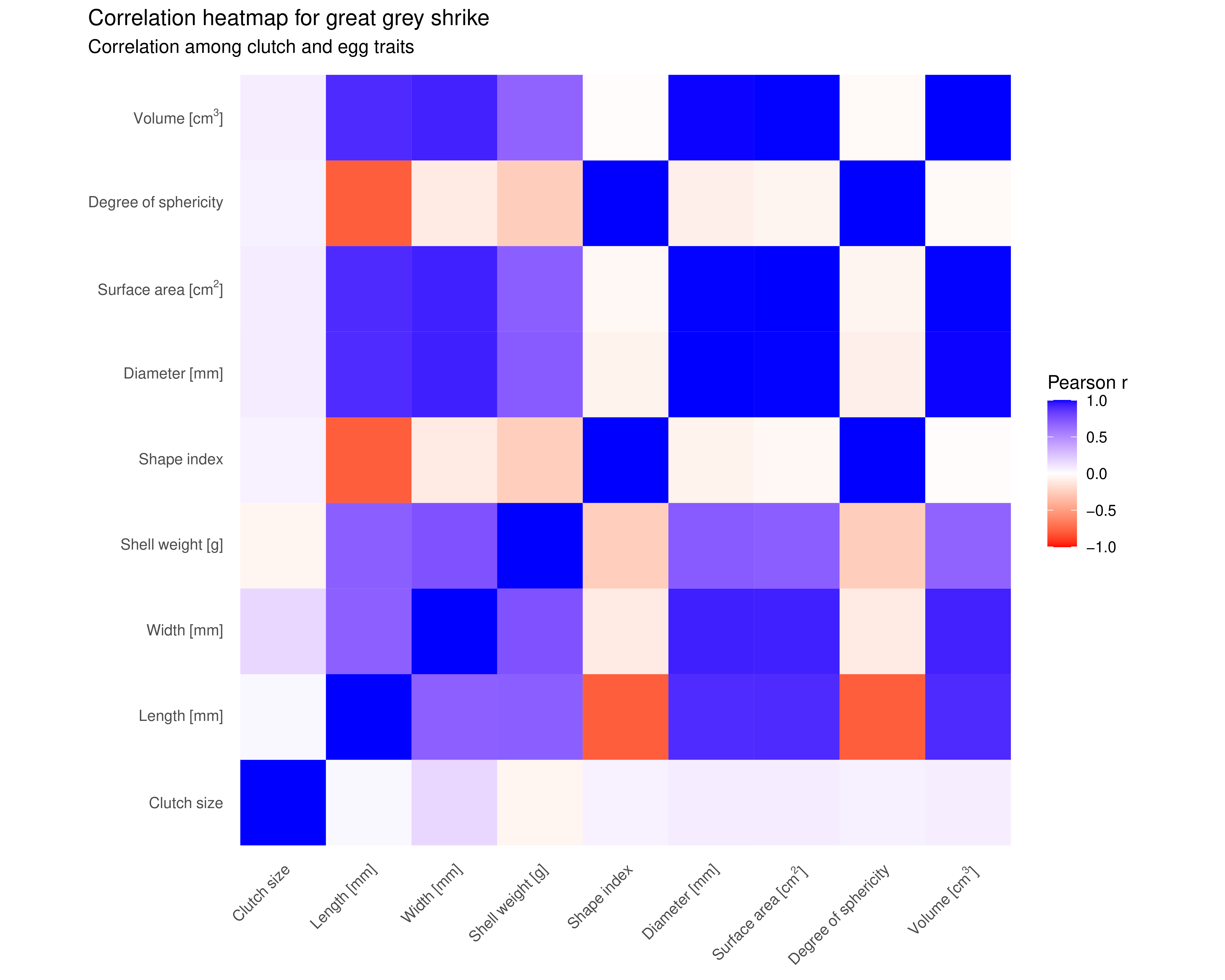

Supplement: Supplementary file 1 — Figure S1: Boxplots showing variation in mean egg traits (length, width, shell weight, shape index, diameter, surface area, degree of sphericity and volume) among shrike species: red‐backed shrike, woodchat shrike, lesser grey shrike and great grey shrike. Each box represents the interquartile range (IQR) with the median indicated by a horizontal line, whiskers extending to 1.5× IQR, and outliers shown as individual points. Figure S2: Among‐year variation in egg morphology traits for four shrike species, red‐backed shrike, woodchat shrike, lesser grey shrike and great grey shrike, based on historical museum collections spanning 1888–1973. Each panel presents non‐parametric comparisons (Kruskal–Wallis test followed by Dunn's pairwise post hoc tests) for key reproductive traits, including egg length, width, shell weight, shape index, volume and clutch size. Boxes show interquartile ranges with medians, whiskers indicate data spread, and letters denote statistically significant differences among years (p < 0.05). p‐values from Kruskal–Wallis tests are provided in each panel. Figure S3: Geographic variation in egg morphology traits of four shrike species, red‐backed shrike, woodchat shrike, lesser grey shrike and great grey shrike, across countries represented in the historical egg collection. Each panel shows mean values (± variation) of key egg traits—including egg length, width, diameter, surface area, volume, shell weight, shape index, degree of sphericity and clutch size—plotted by country. Sample sizes (n) for each country are indicated below the x‐axis. Figure S4: Correlation heatmaps showing relationships among clutch size and mean egg traits (length, width, shell weight, shape index, diameter, surface area, degree of sphericity and volume) across all studied species (ALL) and separately for red‐backed shrike, woodchat shrike, lesser grey shrike and great grey shrike. Colour gradients represent Pearson correlation coefficients ranging from −1 (negative correlati [file ECE3-16-e74065-s001.zip › Figure_S4c.png]

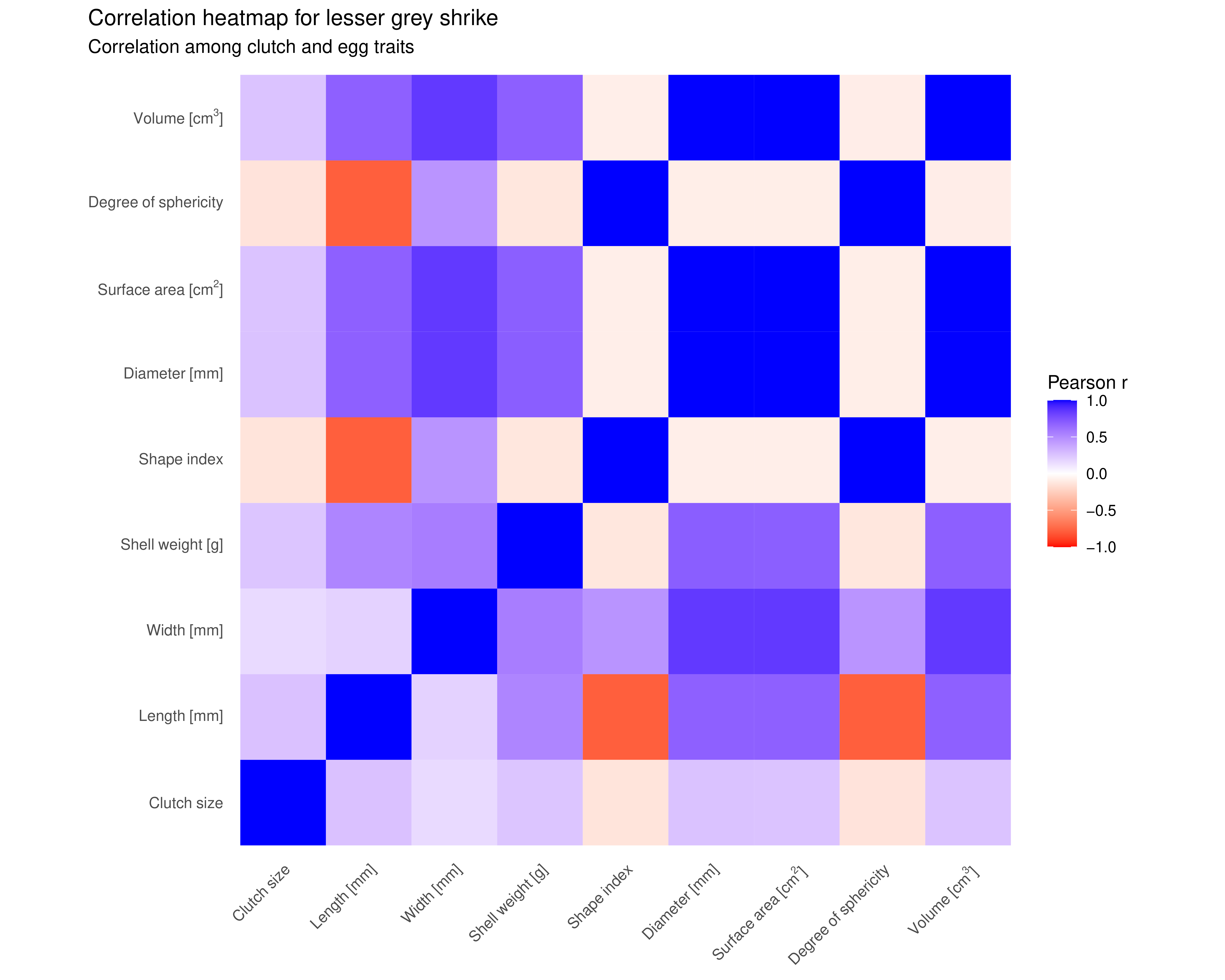

Supplement: Supplementary file 1 — Figure S1: Boxplots showing variation in mean egg traits (length, width, shell weight, shape index, diameter, surface area, degree of sphericity and volume) among shrike species: red‐backed shrike, woodchat shrike, lesser grey shrike and great grey shrike. Each box represents the interquartile range (IQR) with the median indicated by a horizontal line, whiskers extending to 1.5× IQR, and outliers shown as individual points. Figure S2: Among‐year variation in egg morphology traits for four shrike species, red‐backed shrike, woodchat shrike, lesser grey shrike and great grey shrike, based on historical museum collections spanning 1888–1973. Each panel presents non‐parametric comparisons (Kruskal–Wallis test followed by Dunn's pairwise post hoc tests) for key reproductive traits, including egg length, width, shell weight, shape index, volume and clutch size. Boxes show interquartile ranges with medians, whiskers indicate data spread, and letters denote statistically significant differences among years (p < 0.05). p‐values from Kruskal–Wallis tests are provided in each panel. Figure S3: Geographic variation in egg morphology traits of four shrike species, red‐backed shrike, woodchat shrike, lesser grey shrike and great grey shrike, across countries represented in the historical egg collection. Each panel shows mean values (± variation) of key egg traits—including egg length, width, diameter, surface area, volume, shell weight, shape index, degree of sphericity and clutch size—plotted by country. Sample sizes (n) for each country are indicated below the x‐axis. Figure S4: Correlation heatmaps showing relationships among clutch size and mean egg traits (length, width, shell weight, shape index, diameter, surface area, degree of sphericity and volume) across all studied species (ALL) and separately for red‐backed shrike, woodchat shrike, lesser grey shrike and great grey shrike. Colour gradients represent Pearson correlation coefficients ranging from −1 (negative correlati [file ECE3-16-e74065-s001.zip › Figure_S4d.png]

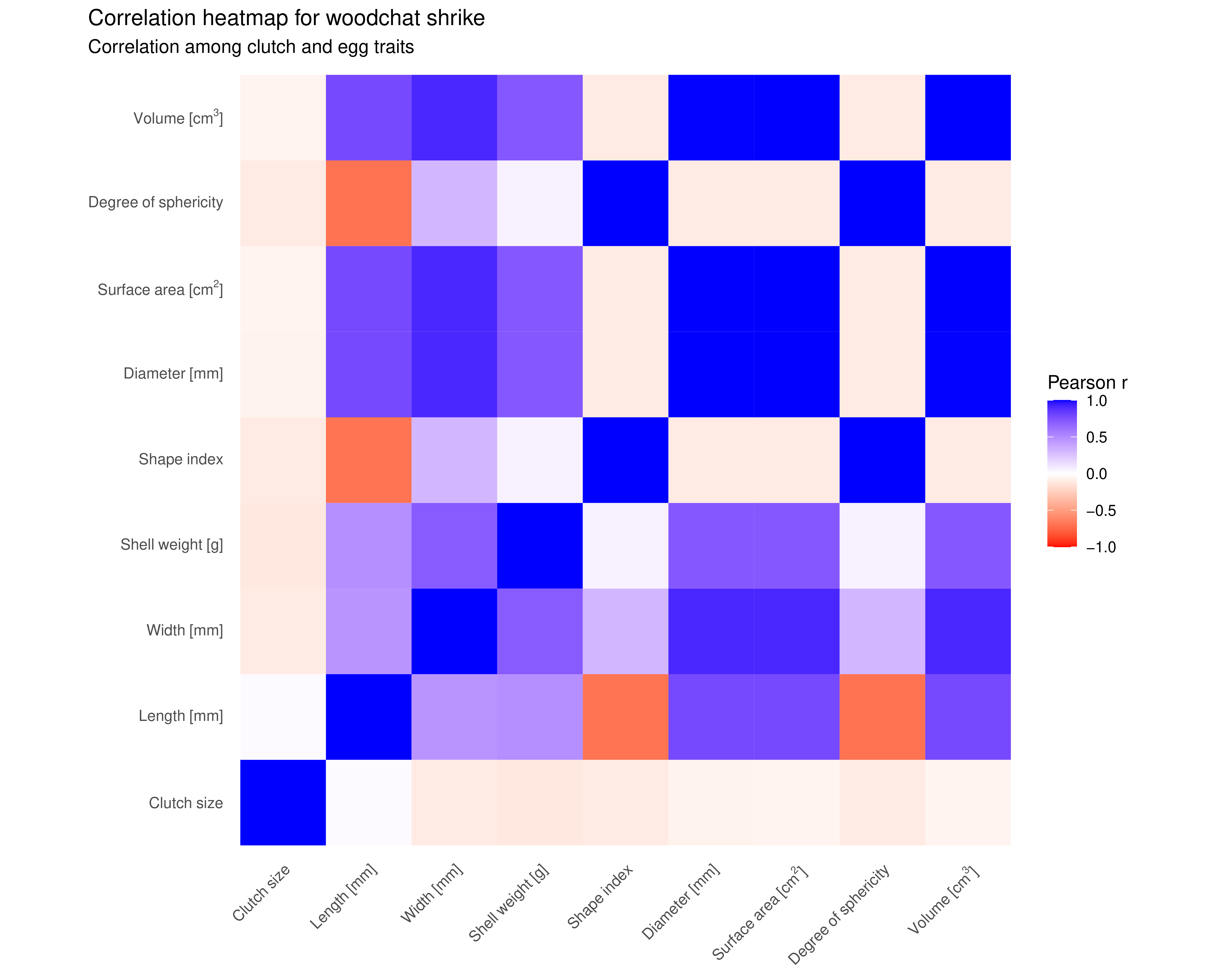

Supplement: Supplementary file 1 — Figure S1: Boxplots showing variation in mean egg traits (length, width, shell weight, shape index, diameter, surface area, degree of sphericity and volume) among shrike species: red‐backed shrike, woodchat shrike, lesser grey shrike and great grey shrike. Each box represents the interquartile range (IQR) with the median indicated by a horizontal line, whiskers extending to 1.5× IQR, and outliers shown as individual points. Figure S2: Among‐year variation in egg morphology traits for four shrike species, red‐backed shrike, woodchat shrike, lesser grey shrike and great grey shrike, based on historical museum collections spanning 1888–1973. Each panel presents non‐parametric comparisons (Kruskal–Wallis test followed by Dunn's pairwise post hoc tests) for key reproductive traits, including egg length, width, shell weight, shape index, volume and clutch size. Boxes show interquartile ranges with medians, whiskers indicate data spread, and letters denote statistically significant differences among years (p < 0.05). p‐values from Kruskal–Wallis tests are provided in each panel. Figure S3: Geographic variation in egg morphology traits of four shrike species, red‐backed shrike, woodchat shrike, lesser grey shrike and great grey shrike, across countries represented in the historical egg collection. Each panel shows mean values (± variation) of key egg traits—including egg length, width, diameter, surface area, volume, shell weight, shape index, degree of sphericity and clutch size—plotted by country. Sample sizes (n) for each country are indicated below the x‐axis. Figure S4: Correlation heatmaps showing relationships among clutch size and mean egg traits (length, width, shell weight, shape index, diameter, surface area, degree of sphericity and volume) across all studied species (ALL) and separately for red‐backed shrike, woodchat shrike, lesser grey shrike and great grey shrike. Colour gradients represent Pearson correlation coefficients ranging from −1 (negative correlati [file ECE3-16-e74065-s001.zip › Figure_S4e.png]

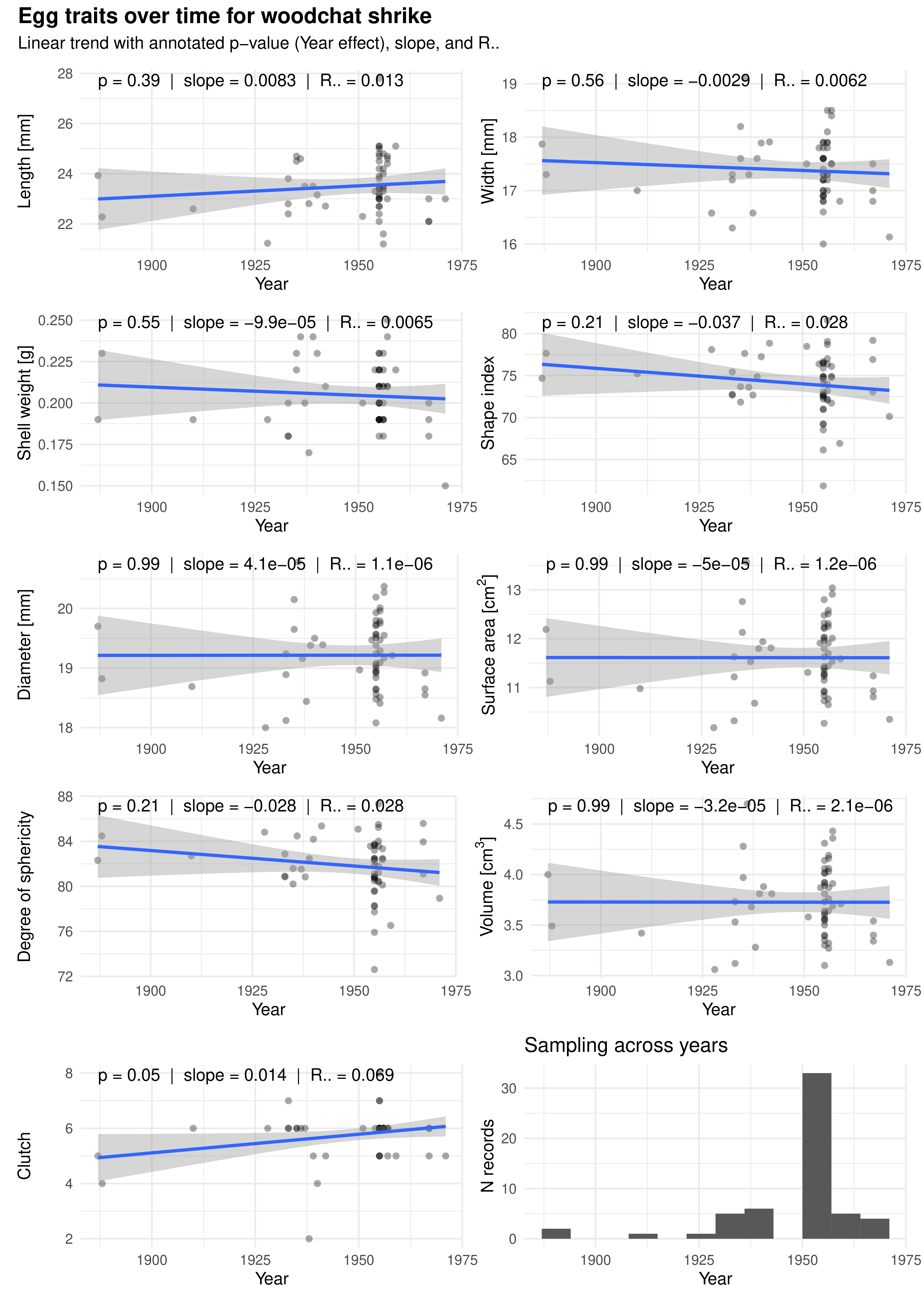

Supplement: Supplementary file 1 — Figure S1: Boxplots showing variation in mean egg traits (length, width, shell weight, shape index, diameter, surface area, degree of sphericity and volume) among shrike species: red‐backed shrike, woodchat shrike, lesser grey shrike and great grey shrike. Each box represents the interquartile range (IQR) with the median indicated by a horizontal line, whiskers extending to 1.5× IQR, and outliers shown as individual points. Figure S2: Among‐year variation in egg morphology traits for four shrike species, red‐backed shrike, woodchat shrike, lesser grey shrike and great grey shrike, based on historical museum collections spanning 1888–1973. Each panel presents non‐parametric comparisons (Kruskal–Wallis test followed by Dunn's pairwise post hoc tests) for key reproductive traits, including egg length, width, shell weight, shape index, volume and clutch size. Boxes show interquartile ranges with medians, whiskers indicate data spread, and letters denote statistically significant differences among years (p < 0.05). p‐values from Kruskal–Wallis tests are provided in each panel. Figure S3: Geographic variation in egg morphology traits of four shrike species, red‐backed shrike, woodchat shrike, lesser grey shrike and great grey shrike, across countries represented in the historical egg collection. Each panel shows mean values (± variation) of key egg traits—including egg length, width, diameter, surface area, volume, shell weight, shape index, degree of sphericity and clutch size—plotted by country. Sample sizes (n) for each country are indicated below the x‐axis. Figure S4: Correlation heatmaps showing relationships among clutch size and mean egg traits (length, width, shell weight, shape index, diameter, surface area, degree of sphericity and volume) across all studied species (ALL) and separately for red‐backed shrike, woodchat shrike, lesser grey shrike and great grey shrike. Colour gradients represent Pearson correlation coefficients ranging from −1 (negative correlati [file ECE3-16-e74065-s001.zip › Figure_S1c.png]

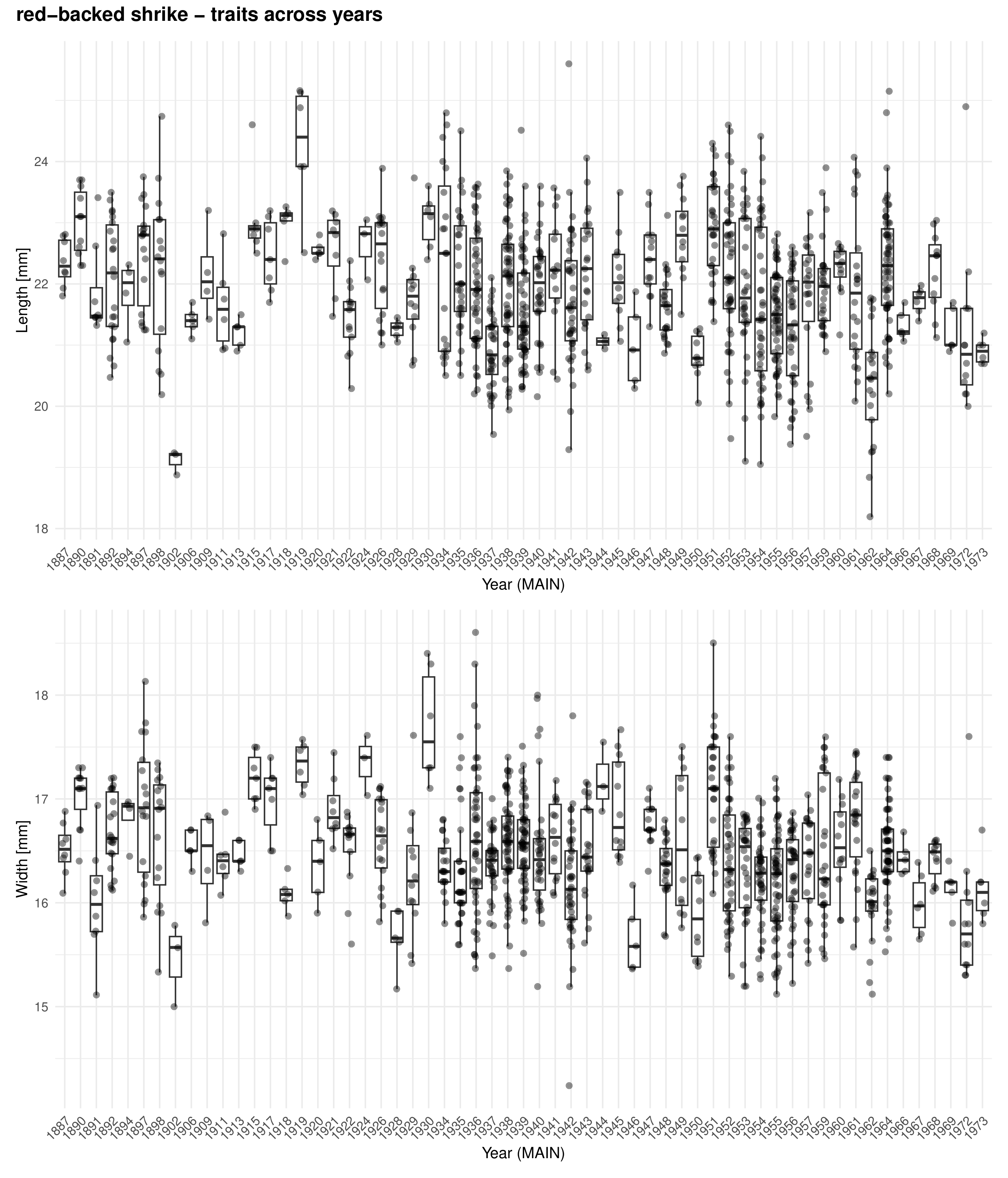

Supplement: Supplementary file 1 — Figure S1: Boxplots showing variation in mean egg traits (length, width, shell weight, shape index, diameter, surface area, degree of sphericity and volume) among shrike species: red‐backed shrike, woodchat shrike, lesser grey shrike and great grey shrike. Each box represents the interquartile range (IQR) with the median indicated by a horizontal line, whiskers extending to 1.5× IQR, and outliers shown as individual points. Figure S2: Among‐year variation in egg morphology traits for four shrike species, red‐backed shrike, woodchat shrike, lesser grey shrike and great grey shrike, based on historical museum collections spanning 1888–1973. Each panel presents non‐parametric comparisons (Kruskal–Wallis test followed by Dunn's pairwise post hoc tests) for key reproductive traits, including egg length, width, shell weight, shape index, volume and clutch size. Boxes show interquartile ranges with medians, whiskers indicate data spread, and letters denote statistically significant differences among years (p < 0.05). p‐values from Kruskal–Wallis tests are provided in each panel. Figure S3: Geographic variation in egg morphology traits of four shrike species, red‐backed shrike, woodchat shrike, lesser grey shrike and great grey shrike, across countries represented in the historical egg collection. Each panel shows mean values (± variation) of key egg traits—including egg length, width, diameter, surface area, volume, shell weight, shape index, degree of sphericity and clutch size—plotted by country. Sample sizes (n) for each country are indicated below the x‐axis. Figure S4: Correlation heatmaps showing relationships among clutch size and mean egg traits (length, width, shell weight, shape index, diameter, surface area, degree of sphericity and volume) across all studied species (ALL) and separately for red‐backed shrike, woodchat shrike, lesser grey shrike and great grey shrike. Colour gradients represent Pearson correlation coefficients ranging from −1 (negative correlati [file ECE3-16-e74065-s001.zip › Figure_S2a.png]

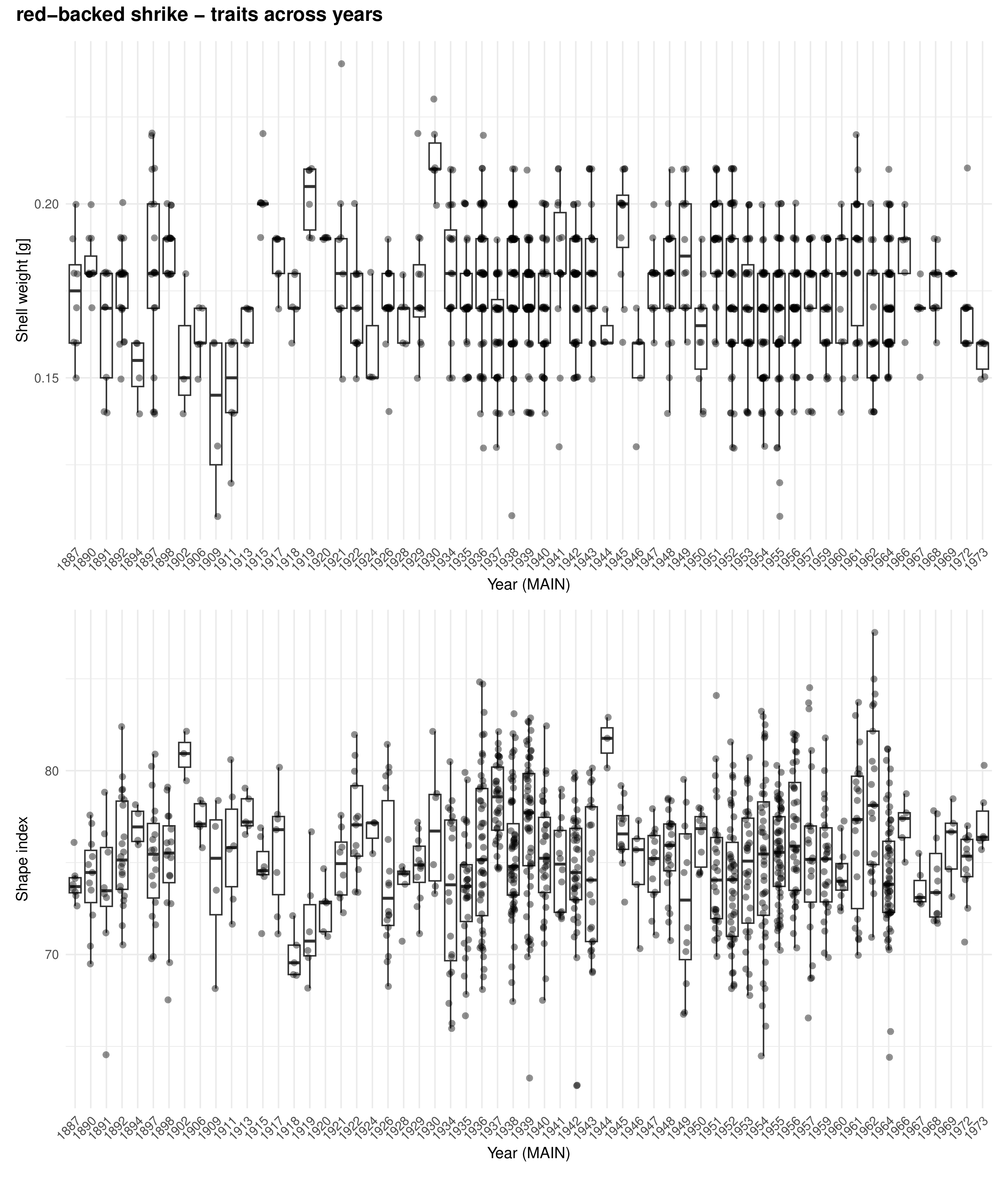

Supplement: Supplementary file 1 — Figure S1: Boxplots showing variation in mean egg traits (length, width, shell weight, shape index, diameter, surface area, degree of sphericity and volume) among shrike species: red‐backed shrike, woodchat shrike, lesser grey shrike and great grey shrike. Each box represents the interquartile range (IQR) with the median indicated by a horizontal line, whiskers extending to 1.5× IQR, and outliers shown as individual points. Figure S2: Among‐year variation in egg morphology traits for four shrike species, red‐backed shrike, woodchat shrike, lesser grey shrike and great grey shrike, based on historical museum collections spanning 1888–1973. Each panel presents non‐parametric comparisons (Kruskal–Wallis test followed by Dunn's pairwise post hoc tests) for key reproductive traits, including egg length, width, shell weight, shape index, volume and clutch size. Boxes show interquartile ranges with medians, whiskers indicate data spread, and letters denote statistically significant differences among years (p < 0.05). p‐values from Kruskal–Wallis tests are provided in each panel. Figure S3: Geographic variation in egg morphology traits of four shrike species, red‐backed shrike, woodchat shrike, lesser grey shrike and great grey shrike, across countries represented in the historical egg collection. Each panel shows mean values (± variation) of key egg traits—including egg length, width, diameter, surface area, volume, shell weight, shape index, degree of sphericity and clutch size—plotted by country. Sample sizes (n) for each country are indicated below the x‐axis. Figure S4: Correlation heatmaps showing relationships among clutch size and mean egg traits (length, width, shell weight, shape index, diameter, surface area, degree of sphericity and volume) across all studied species (ALL) and separately for red‐backed shrike, woodchat shrike, lesser grey shrike and great grey shrike. Colour gradients represent Pearson correlation coefficients ranging from −1 (negative correlati [file ECE3-16-e74065-s001.zip › Figure_S2b.png]

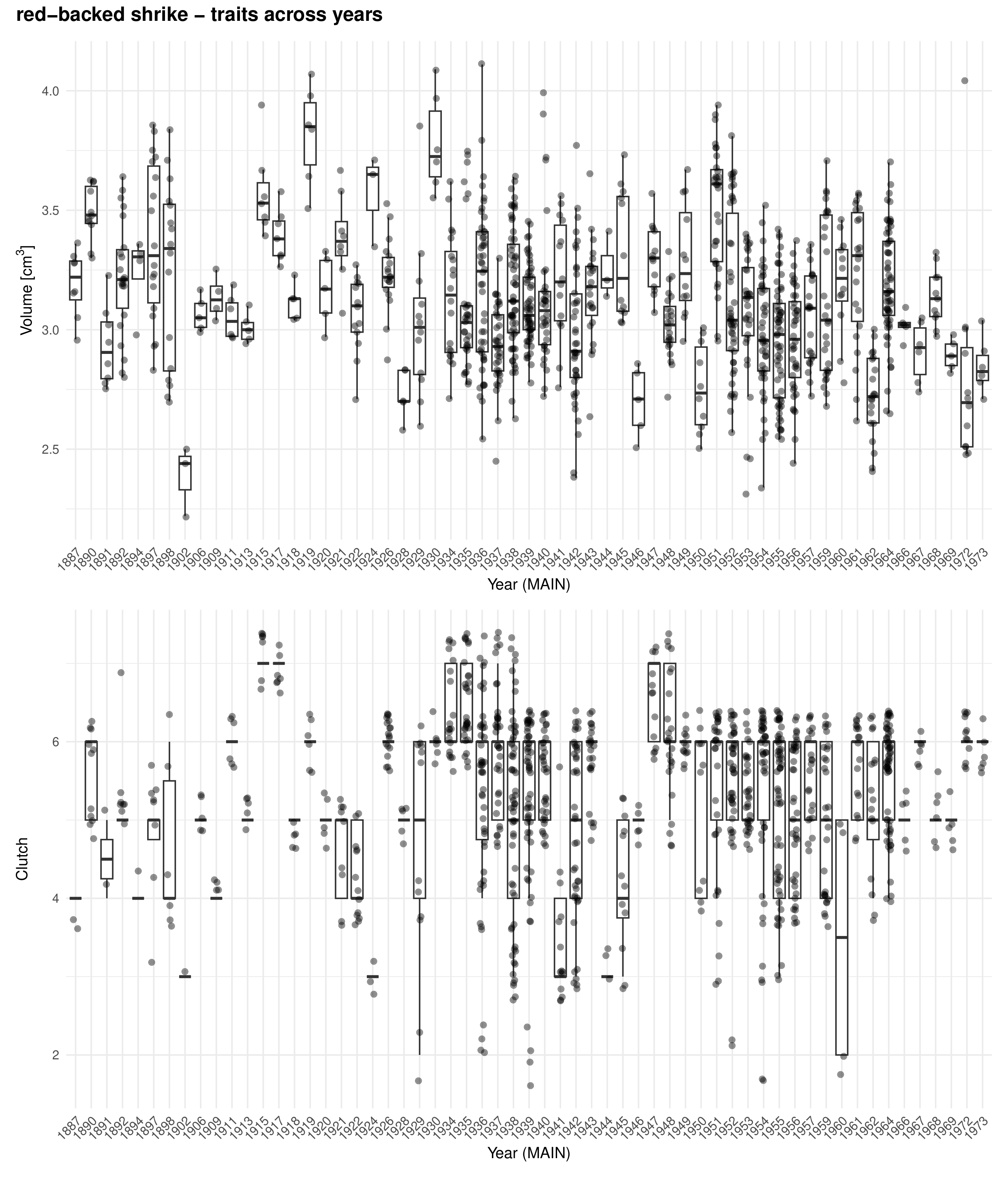

Supplement: Supplementary file 1 — Figure S1: Boxplots showing variation in mean egg traits (length, width, shell weight, shape index, diameter, surface area, degree of sphericity and volume) among shrike species: red‐backed shrike, woodchat shrike, lesser grey shrike and great grey shrike. Each box represents the interquartile range (IQR) with the median indicated by a horizontal line, whiskers extending to 1.5× IQR, and outliers shown as individual points. Figure S2: Among‐year variation in egg morphology traits for four shrike species, red‐backed shrike, woodchat shrike, lesser grey shrike and great grey shrike, based on historical museum collections spanning 1888–1973. Each panel presents non‐parametric comparisons (Kruskal–Wallis test followed by Dunn's pairwise post hoc tests) for key reproductive traits, including egg length, width, shell weight, shape index, volume and clutch size. Boxes show interquartile ranges with medians, whiskers indicate data spread, and letters denote statistically significant differences among years (p < 0.05). p‐values from Kruskal–Wallis tests are provided in each panel. Figure S3: Geographic variation in egg morphology traits of four shrike species, red‐backed shrike, woodchat shrike, lesser grey shrike and great grey shrike, across countries represented in the historical egg collection. Each panel shows mean values (± variation) of key egg traits—including egg length, width, diameter, surface area, volume, shell weight, shape index, degree of sphericity and clutch size—plotted by country. Sample sizes (n) for each country are indicated below the x‐axis. Figure S4: Correlation heatmaps showing relationships among clutch size and mean egg traits (length, width, shell weight, shape index, diameter, surface area, degree of sphericity and volume) across all studied species (ALL) and separately for red‐backed shrike, woodchat shrike, lesser grey shrike and great grey shrike. Colour gradients represent Pearson correlation coefficients ranging from −1 (negative correlati [file ECE3-16-e74065-s001.zip › Figure_S2c.png]

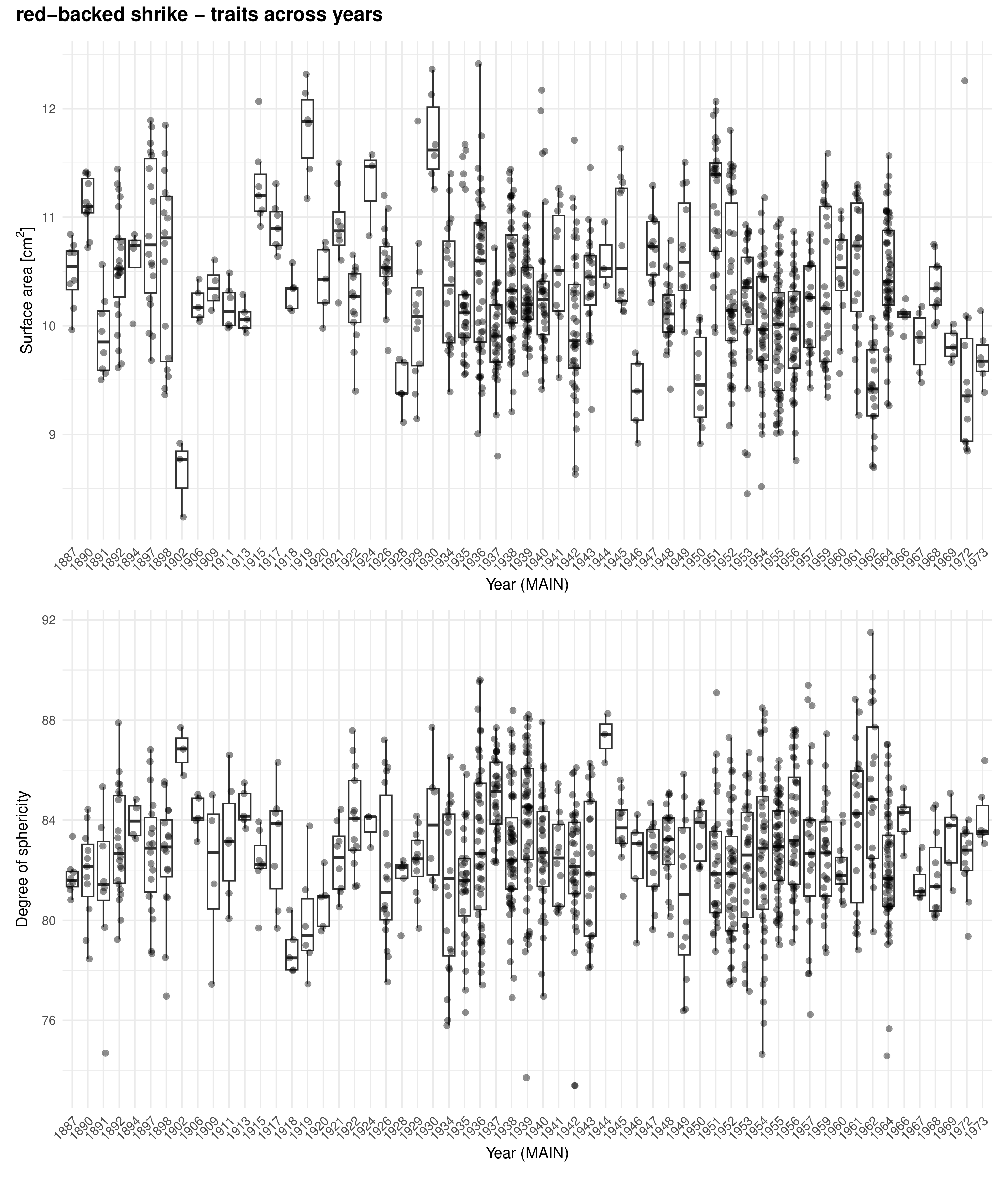

Supplement: Supplementary file 1 — Figure S1: Boxplots showing variation in mean egg traits (length, width, shell weight, shape index, diameter, surface area, degree of sphericity and volume) among shrike species: red‐backed shrike, woodchat shrike, lesser grey shrike and great grey shrike. Each box represents the interquartile range (IQR) with the median indicated by a horizontal line, whiskers extending to 1.5× IQR, and outliers shown as individual points. Figure S2: Among‐year variation in egg morphology traits for four shrike species, red‐backed shrike, woodchat shrike, lesser grey shrike and great grey shrike, based on historical museum collections spanning 1888–1973. Each panel presents non‐parametric comparisons (Kruskal–Wallis test followed by Dunn's pairwise post hoc tests) for key reproductive traits, including egg length, width, shell weight, shape index, volume and clutch size. Boxes show interquartile ranges with medians, whiskers indicate data spread, and letters denote statistically significant differences among years (p < 0.05). p‐values from Kruskal–Wallis tests are provided in each panel. Figure S3: Geographic variation in egg morphology traits of four shrike species, red‐backed shrike, woodchat shrike, lesser grey shrike and great grey shrike, across countries represented in the historical egg collection. Each panel shows mean values (± variation) of key egg traits—including egg length, width, diameter, surface area, volume, shell weight, shape index, degree of sphericity and clutch size—plotted by country. Sample sizes (n) for each country are indicated below the x‐axis. Figure S4: Correlation heatmaps showing relationships among clutch size and mean egg traits (length, width, shell weight, shape index, diameter, surface area, degree of sphericity and volume) across all studied species (ALL) and separately for red‐backed shrike, woodchat shrike, lesser grey shrike and great grey shrike. Colour gradients represent Pearson correlation coefficients ranging from −1 (negative correlati [file ECE3-16-e74065-s001.zip › Figure_S2d.png]

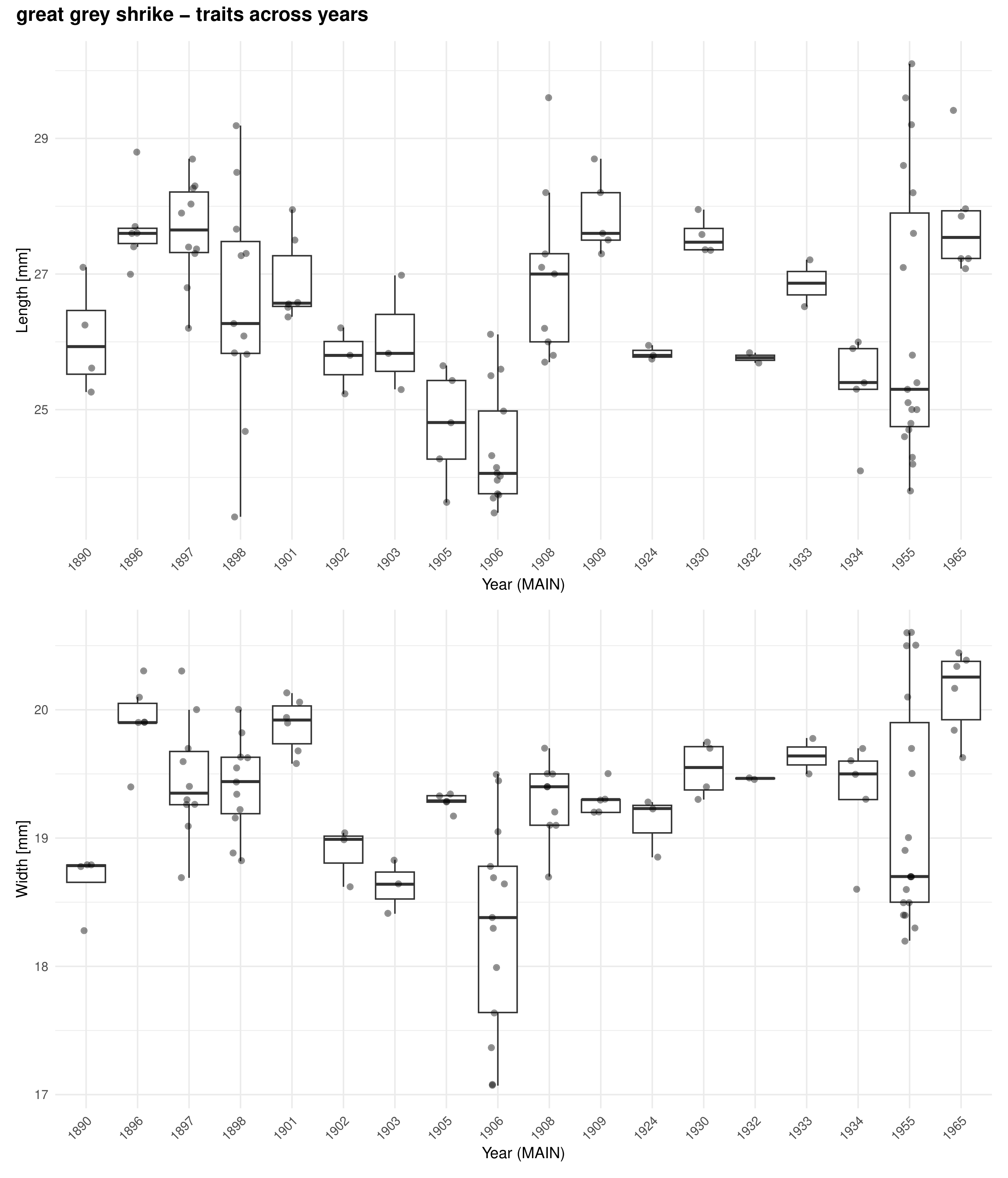

Supplement: Supplementary file 1 — Figure S1: Boxplots showing variation in mean egg traits (length, width, shell weight, shape index, diameter, surface area, degree of sphericity and volume) among shrike species: red‐backed shrike, woodchat shrike, lesser grey shrike and great grey shrike. Each box represents the interquartile range (IQR) with the median indicated by a horizontal line, whiskers extending to 1.5× IQR, and outliers shown as individual points. Figure S2: Among‐year variation in egg morphology traits for four shrike species, red‐backed shrike, woodchat shrike, lesser grey shrike and great grey shrike, based on historical museum collections spanning 1888–1973. Each panel presents non‐parametric comparisons (Kruskal–Wallis test followed by Dunn's pairwise post hoc tests) for key reproductive traits, including egg length, width, shell weight, shape index, volume and clutch size. Boxes show interquartile ranges with medians, whiskers indicate data spread, and letters denote statistically significant differences among years (p < 0.05). p‐values from Kruskal–Wallis tests are provided in each panel. Figure S3: Geographic variation in egg morphology traits of four shrike species, red‐backed shrike, woodchat shrike, lesser grey shrike and great grey shrike, across countries represented in the historical egg collection. Each panel shows mean values (± variation) of key egg traits—including egg length, width, diameter, surface area, volume, shell weight, shape index, degree of sphericity and clutch size—plotted by country. Sample sizes (n) for each country are indicated below the x‐axis. Figure S4: Correlation heatmaps showing relationships among clutch size and mean egg traits (length, width, shell weight, shape index, diameter, surface area, degree of sphericity and volume) across all studied species (ALL) and separately for red‐backed shrike, woodchat shrike, lesser grey shrike and great grey shrike. Colour gradients represent Pearson correlation coefficients ranging from −1 (negative correlati [file ECE3-16-e74065-s001.zip › Figure_S2e.png]

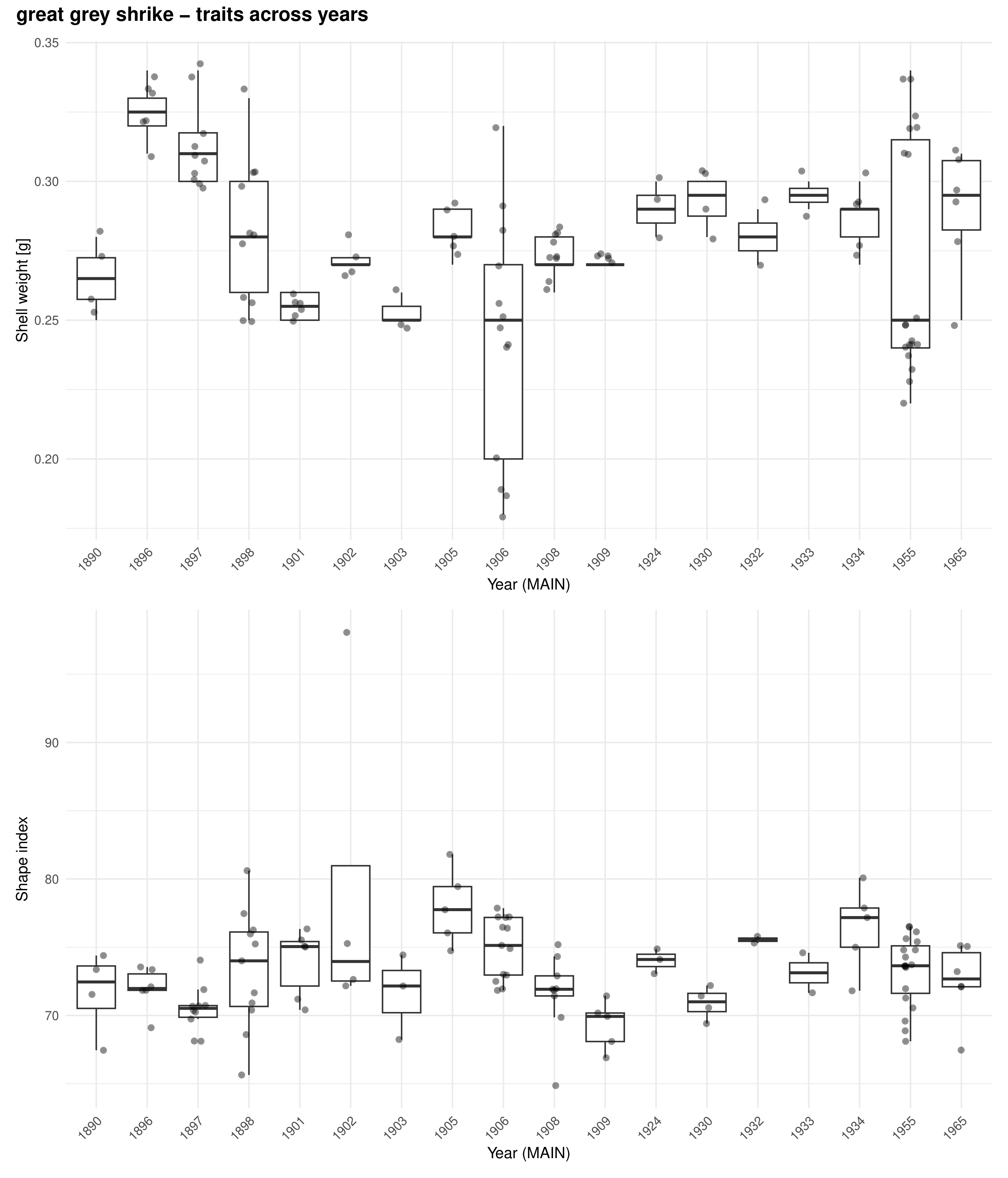

Supplement: Supplementary file 1 — Figure S1: Boxplots showing variation in mean egg traits (length, width, shell weight, shape index, diameter, surface area, degree of sphericity and volume) among shrike species: red‐backed shrike, woodchat shrike, lesser grey shrike and great grey shrike. Each box represents the interquartile range (IQR) with the median indicated by a horizontal line, whiskers extending to 1.5× IQR, and outliers shown as individual points. Figure S2: Among‐year variation in egg morphology traits for four shrike species, red‐backed shrike, woodchat shrike, lesser grey shrike and great grey shrike, based on historical museum collections spanning 1888–1973. Each panel presents non‐parametric comparisons (Kruskal–Wallis test followed by Dunn's pairwise post hoc tests) for key reproductive traits, including egg length, width, shell weight, shape index, volume and clutch size. Boxes show interquartile ranges with medians, whiskers indicate data spread, and letters denote statistically significant differences among years (p < 0.05). p‐values from Kruskal–Wallis tests are provided in each panel. Figure S3: Geographic variation in egg morphology traits of four shrike species, red‐backed shrike, woodchat shrike, lesser grey shrike and great grey shrike, across countries represented in the historical egg collection. Each panel shows mean values (± variation) of key egg traits—including egg length, width, diameter, surface area, volume, shell weight, shape index, degree of sphericity and clutch size—plotted by country. Sample sizes (n) for each country are indicated below the x‐axis. Figure S4: Correlation heatmaps showing relationships among clutch size and mean egg traits (length, width, shell weight, shape index, diameter, surface area, degree of sphericity and volume) across all studied species (ALL) and separately for red‐backed shrike, woodchat shrike, lesser grey shrike and great grey shrike. Colour gradients represent Pearson correlation coefficients ranging from −1 (negative correlati [file ECE3-16-e74065-s001.zip › Figure_S2f.png]

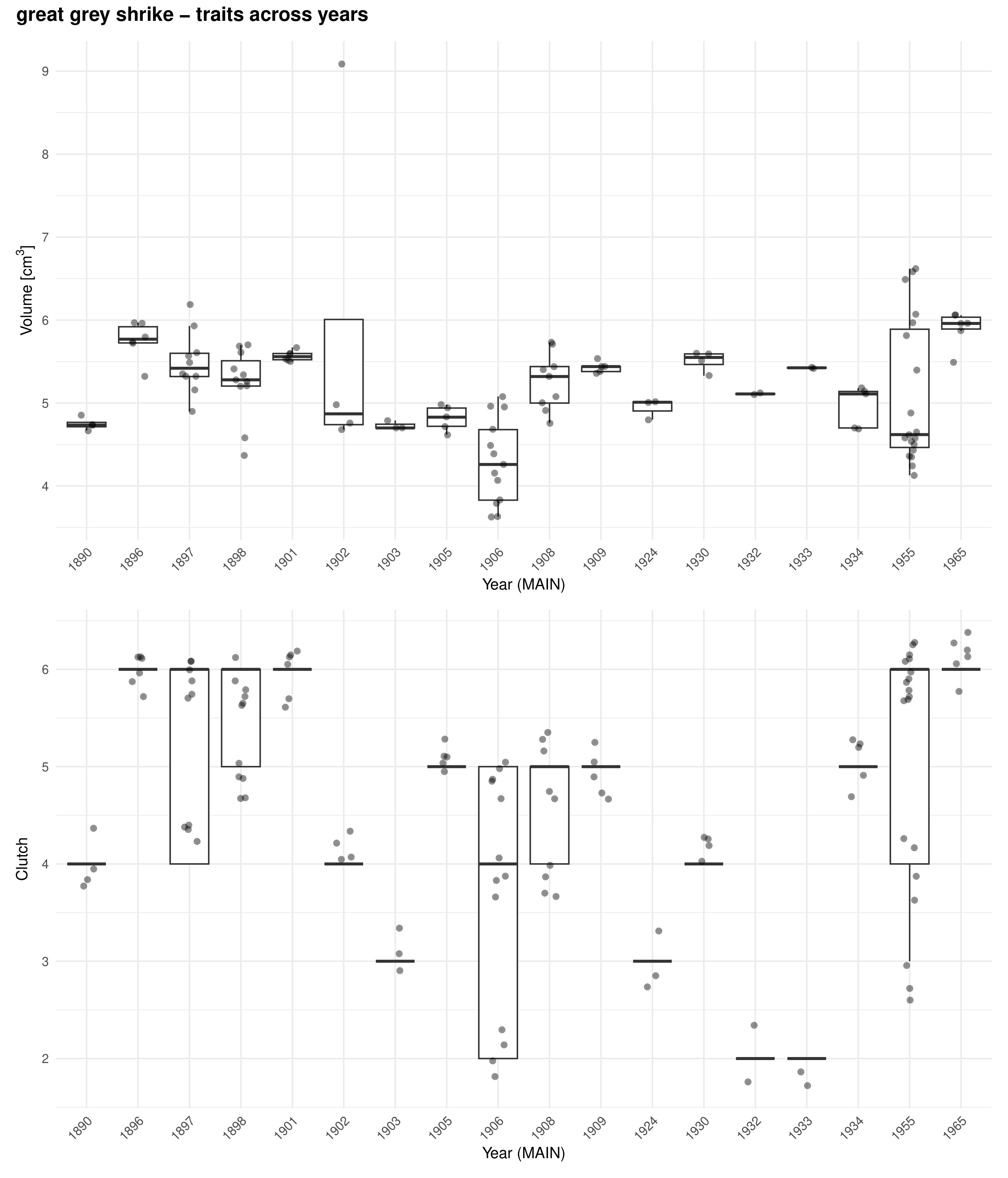

Supplement: Supplementary file 1 — Figure S1: Boxplots showing variation in mean egg traits (length, width, shell weight, shape index, diameter, surface area, degree of sphericity and volume) among shrike species: red‐backed shrike, woodchat shrike, lesser grey shrike and great grey shrike. Each box represents the interquartile range (IQR) with the median indicated by a horizontal line, whiskers extending to 1.5× IQR, and outliers shown as individual points. Figure S2: Among‐year variation in egg morphology traits for four shrike species, red‐backed shrike, woodchat shrike, lesser grey shrike and great grey shrike, based on historical museum collections spanning 1888–1973. Each panel presents non‐parametric comparisons (Kruskal–Wallis test followed by Dunn's pairwise post hoc tests) for key reproductive traits, including egg length, width, shell weight, shape index, volume and clutch size. Boxes show interquartile ranges with medians, whiskers indicate data spread, and letters denote statistically significant differences among years (p < 0.05). p‐values from Kruskal–Wallis tests are provided in each panel. Figure S3: Geographic variation in egg morphology traits of four shrike species, red‐backed shrike, woodchat shrike, lesser grey shrike and great grey shrike, across countries represented in the historical egg collection. Each panel shows mean values (± variation) of key egg traits—including egg length, width, diameter, surface area, volume, shell weight, shape index, degree of sphericity and clutch size—plotted by country. Sample sizes (n) for each country are indicated below the x‐axis. Figure S4: Correlation heatmaps showing relationships among clutch size and mean egg traits (length, width, shell weight, shape index, diameter, surface area, degree of sphericity and volume) across all studied species (ALL) and separately for red‐backed shrike, woodchat shrike, lesser grey shrike and great grey shrike. Colour gradients represent Pearson correlation coefficients ranging from −1 (negative correlati [file ECE3-16-e74065-s001.zip › Figure_S2g.png]

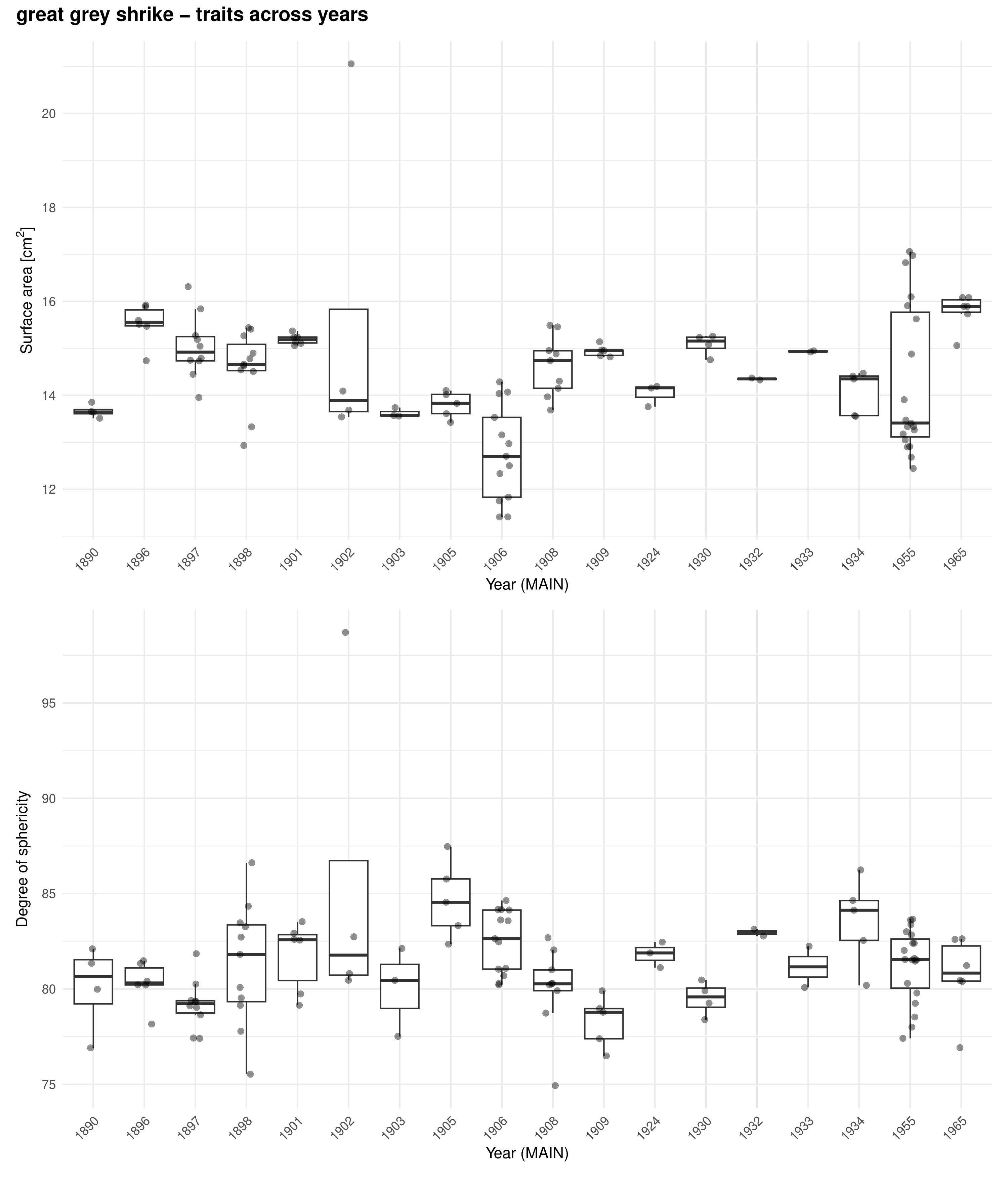

Supplement: Supplementary file 1 — Figure S1: Boxplots showing variation in mean egg traits (length, width, shell weight, shape index, diameter, surface area, degree of sphericity and volume) among shrike species: red‐backed shrike, woodchat shrike, lesser grey shrike and great grey shrike. Each box represents the interquartile range (IQR) with the median indicated by a horizontal line, whiskers extending to 1.5× IQR, and outliers shown as individual points. Figure S2: Among‐year variation in egg morphology traits for four shrike species, red‐backed shrike, woodchat shrike, lesser grey shrike and great grey shrike, based on historical museum collections spanning 1888–1973. Each panel presents non‐parametric comparisons (Kruskal–Wallis test followed by Dunn's pairwise post hoc tests) for key reproductive traits, including egg length, width, shell weight, shape index, volume and clutch size. Boxes show interquartile ranges with medians, whiskers indicate data spread, and letters denote statistically significant differences among years (p < 0.05). p‐values from Kruskal–Wallis tests are provided in each panel. Figure S3: Geographic variation in egg morphology traits of four shrike species, red‐backed shrike, woodchat shrike, lesser grey shrike and great grey shrike, across countries represented in the historical egg collection. Each panel shows mean values (± variation) of key egg traits—including egg length, width, diameter, surface area, volume, shell weight, shape index, degree of sphericity and clutch size—plotted by country. Sample sizes (n) for each country are indicated below the x‐axis. Figure S4: Correlation heatmaps showing relationships among clutch size and mean egg traits (length, width, shell weight, shape index, diameter, surface area, degree of sphericity and volume) across all studied species (ALL) and separately for red‐backed shrike, woodchat shrike, lesser grey shrike and great grey shrike. Colour gradients represent Pearson correlation coefficients ranging from −1 (negative correlati [file ECE3-16-e74065-s001.zip › Figure_S2h.png]

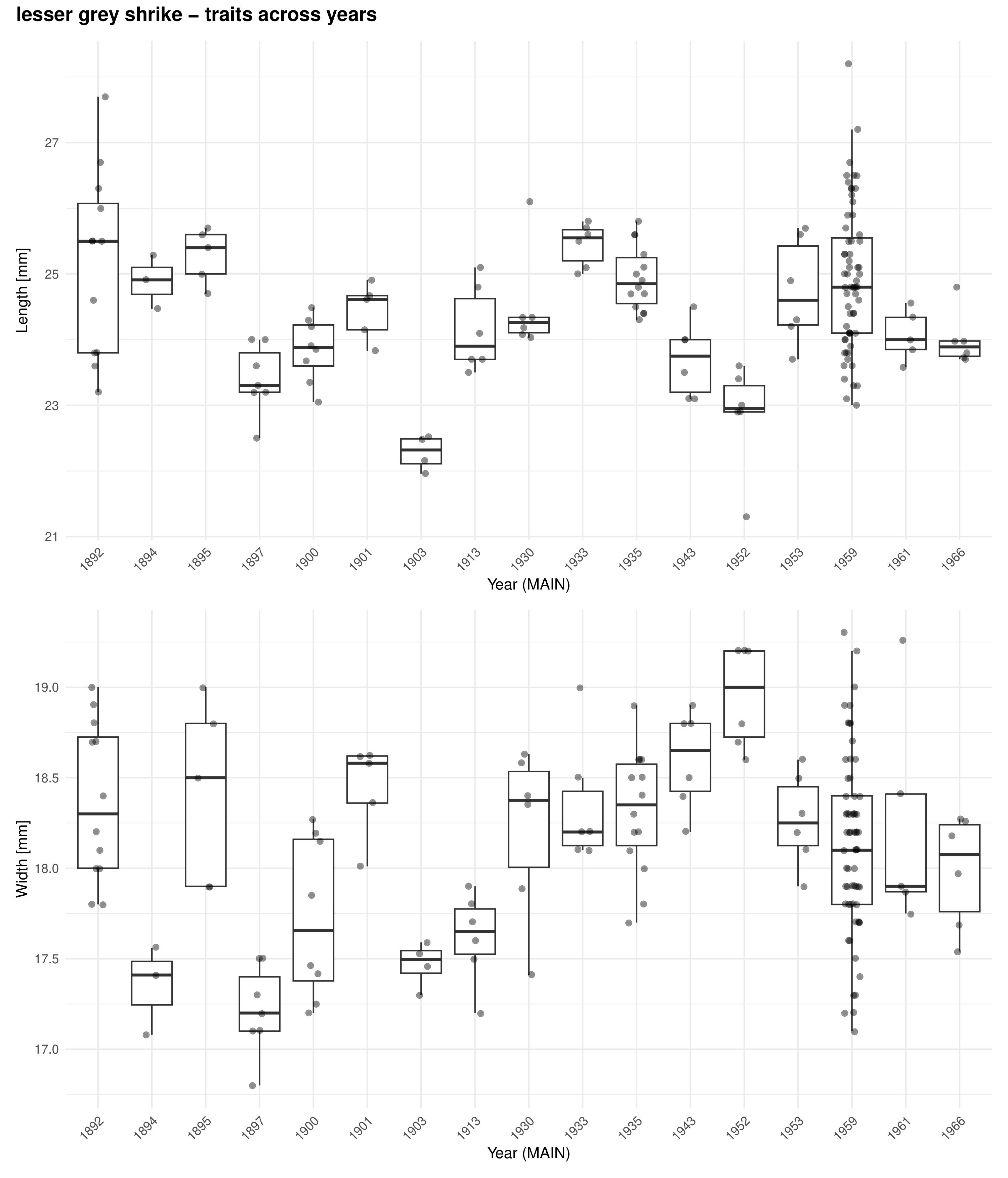

Supplement: Supplementary file 1 — Figure S1: Boxplots showing variation in mean egg traits (length, width, shell weight, shape index, diameter, surface area, degree of sphericity and volume) among shrike species: red‐backed shrike, woodchat shrike, lesser grey shrike and great grey shrike. Each box represents the interquartile range (IQR) with the median indicated by a horizontal line, whiskers extending to 1.5× IQR, and outliers shown as individual points. Figure S2: Among‐year variation in egg morphology traits for four shrike species, red‐backed shrike, woodchat shrike, lesser grey shrike and great grey shrike, based on historical museum collections spanning 1888–1973. Each panel presents non‐parametric comparisons (Kruskal–Wallis test followed by Dunn's pairwise post hoc tests) for key reproductive traits, including egg length, width, shell weight, shape index, volume and clutch size. Boxes show interquartile ranges with medians, whiskers indicate data spread, and letters denote statistically significant differences among years (p < 0.05). p‐values from Kruskal–Wallis tests are provided in each panel. Figure S3: Geographic variation in egg morphology traits of four shrike species, red‐backed shrike, woodchat shrike, lesser grey shrike and great grey shrike, across countries represented in the historical egg collection. Each panel shows mean values (± variation) of key egg traits—including egg length, width, diameter, surface area, volume, shell weight, shape index, degree of sphericity and clutch size—plotted by country. Sample sizes (n) for each country are indicated below the x‐axis. Figure S4: Correlation heatmaps showing relationships among clutch size and mean egg traits (length, width, shell weight, shape index, diameter, surface area, degree of sphericity and volume) across all studied species (ALL) and separately for red‐backed shrike, woodchat shrike, lesser grey shrike and great grey shrike. Colour gradients represent Pearson correlation coefficients ranging from −1 (negative correlati [file ECE3-16-e74065-s001.zip › Figure_S2i.png]
